# NLRP3 inflammasome–driven hemophagocytic lymphohistiocytosis occurs independent of IL-1β and IL-18 and is targetable by BET inhibitors

**DOI:** 10.1126/sciadv.adv0079

**Published:** 2025-07-09

**Authors:** Farzaneh Shojaee, Esmaeel Azadian, Min Xian Wong, Xiuquan Ma, James Rickard, Jiyi Pang, Catherine Hall, Andrew J. Kueh, Seth L. Masters, Inmaculada Rioja, Rab K. Prinjha, Marcel Doerflinger, Kate E. Lawlor, Maryam Rashidi, James E. Vince

**Affiliations:** ^1^ The Walter and Eliza Hall Institute of Medical Research, Parkville 3052, Australia.; ^2^ Department of Medical Biology, University of Melbourne, Parkville 3010, Australia.; ^3^ Department of Biological Sciences, National University of Singapore, Singapore 117558, Singapore.; ^4^ Dorevitch Pathology, Heidelberg 3084, Australia.; ^5^ Olivia Newton-John Cancer and Wellness Centre, Austin Health, Heidelberg 3084, Australia.; ^6^ School of Cancer Medicine, La Trobe University, Heidelberg 3084, Australia.; ^7^ Centre for Innate Immunity and Infectious Diseases, Hudson Institute of Medical Research, Clayton 3168, Australia.; ^8^ Department of Molecular and Translational Science, Monash University, Clayton 3168, Australia.; ^9^ BicycleTx Limited, Portway Building, Granta Park, Cambridge CB21 6GS, UK.; ^10^ Curve Therapeutics, Delta House, Enterprise Road, Southampton Science Park, Southampton SO16 7NS, UK.

## Abstract

Hemophagocytic lymphohistiocytosis (HLH) is a potentially fatal cytokine storm syndrome. Its high mortality rate reflects limited therapeutic options and a poor understanding of disease-causing signaling. We show that the NLRP3 inflammasome is responsible for increased mortality in a model of secondary HLH (sHLH). Unexpectedly, neither deletion of the NLRP3-activated pyroptotic effector GSDMD nor combined deletion of the inflammasome-activated cytokines interleukin-1β (IL-1β) and IL-18 conferred strong protection from sHLH. Instead, co-deletion of GSDMD and caspase-8–activated GSDME limited sHLH-driven lethality, demonstrating redundancy in the pyroptotic machinery required to induce sHLH. We also found that bromodomain and extraterminal domain (BET) inhibitors prevent NLRP3-driven pyroptosis, which acted by blocking inflammasome priming. BET inhibitors prevented increased NLRP3 levels in diseased tissue, limited the production of sHLH-associated IL-1β, interferon-γ, and tumor necrosis factor, and protected from sHLH pathogenesis. These findings suggest that targeting NLRP3 could limit sHLH and identify clinically relevant bromodomain-selective BET inhibitors capable of eliminating NLRP3-driven pyroptosis and the sHLH cytokine storm.

## Teaser

Targeting NLRP3 with BET inhibitors limits disease in the cytokine storm syndrome, secondary hemophagocytic lymphohistiocytosis.

## INTRODUCTION

Hemophagocytic lymphohistiocytosis (HLH) and the related entity macrophage activation syndrome are cytokine storm syndromes that result from immune cell–driven hyperinflammatory responses, which frequently result in multiorgan damage and death (*1*). HLH is classified into primary (familial) and secondary (acquired) forms (*2*). Primary HLH is caused by monogenic mutations in genes that impair the function of cytotoxic T cells and natural killer cells, such as *PRF1*, *UNC13D*, *STXBP2*, and *STX11* (*3*, *4*), as well as mutations in *BIRC4* (XIAP), whose loss promotes excess cell death signaling–induced inflammation (*5*–*8*). In contrast, secondary HLH (sHLH) does not necessarily involve known genetic lesions and is triggered by underlying conditions such as viral, protozoan, fungal, or bacterial infections; malignancies such as lymphoma; and autoimmune disorders (*9*). Fever, hepatosplenomegaly, hyperferritinemia, and pancytopenia are common clinical manifestations of HLH, which are accompanied by increased levels of cytokines, such as interferons (IFNs), interleukin-1β (IL-1β), IL-18, IL-6, and tumor necrosis factor (TNF) (*10*–*13*). Despite these well-characterized HLH features and the increased use of targeted therapies (*14*), at the molecular level, the underlying disease-driving signaling pathways are still poorly understood (*15*).

Increased levels of the inflammasome-activated cytokines IL-1β and IL-18 are frequently observed in HLH and macrophage activation syndrome (*12*, *16*, *17*), implicating aberrant inflammasome activity in disease pathogenesis. Both IL-1β and IL-18 are synthesized as inactive precursor proteins upon pathogen ligand sensing by innate immune cells such as macrophages. However, IL-1β and IL-18 are only processed into their bioactive fragments upon the activation and assembly of the cytosolic inflammasome complex, which engages caspase-1 to cleave IL-1β and IL-18, in addition to the pyroptotic cell death effector protein gasdermin D (GSDMD) (*18*, *19*). Cleavage of GSDMD by caspase-1, or via the noncanonical caspase-11 inflammasome, releases the inhibitory GSDMD C-terminal domain, allowing N-terminal GSDMD to form pores in the plasma membrane to cause pyroptosis (*20*, *21*). At the same time, GSDMD pore formation is also important for the efficient release of mature IL-1β into the surrounding environment (*22*). Like GSDMD, the N-terminal domain of other gasdermin family members, including GSDMA, GSDMB, GSDMC, and GSDME, are capable of triggering pyroptotic cell death via the formation of membrane pores (*23*). In particular, the ability of apoptotic caspase-8 to process GSDMD (*24*, *25*), and caspase-3 activation of GSDME (*26*, *27*), highlights cross-talk in apoptotic-pyroptotic cell death signaling and the ability of apoptotic caspases to drive an inflammatory cell death phenotype in a context-specific manner (*28*).

Activating mutations in several inflammasome sensor proteins, such as *NLRP3*, *NLRC4*, and *MEFV*, result in autoinflammatory conditions that respond favorably to the inhibition of IL-1 (*29*), and in the case of *NLRC4* mutations, the clinical course of disease can resemble HLH (*30*, *31*). Emerging evidence suggests that IL-1 blockade should be considered as a treatment for sHLH (*32*), although the high levels of IL-18 (*13*, *17*) highlight it as an additional therapeutic target. Mouse studies show that combined IL-18 and IL-1 blockade confers protection in septic shock models (*33*), and IL-18 targeting has also proven successful in *NLRC4* and *XIAP* mutant patients (*34*, *35*). However, it remains unclear as to which inflammasome sensor protein drives high levels of IL-1β and IL-18 activation in sHLH, whether combined IL-1β and IL-18 targeting would be beneficial, or whether pyroptotic cell death may drive pathology independent of IL-1β and IL-18. Here, using a well-characterized sHLH model, we now show that NLRP3 can promote pyroptotic GSDMD- and GSDME-driven lethality independent of IL-1β and IL-18 and identify bromodomain (BRD)–selective bromo- and extraterminal (BET) inhibitors (BETis) as blockers of NLRP3 responses and consequent sHLH disease severity.

## RESULTS

### The NLRP3 inflammasome can drive disease severity in sHLH

**Fig. 1.**
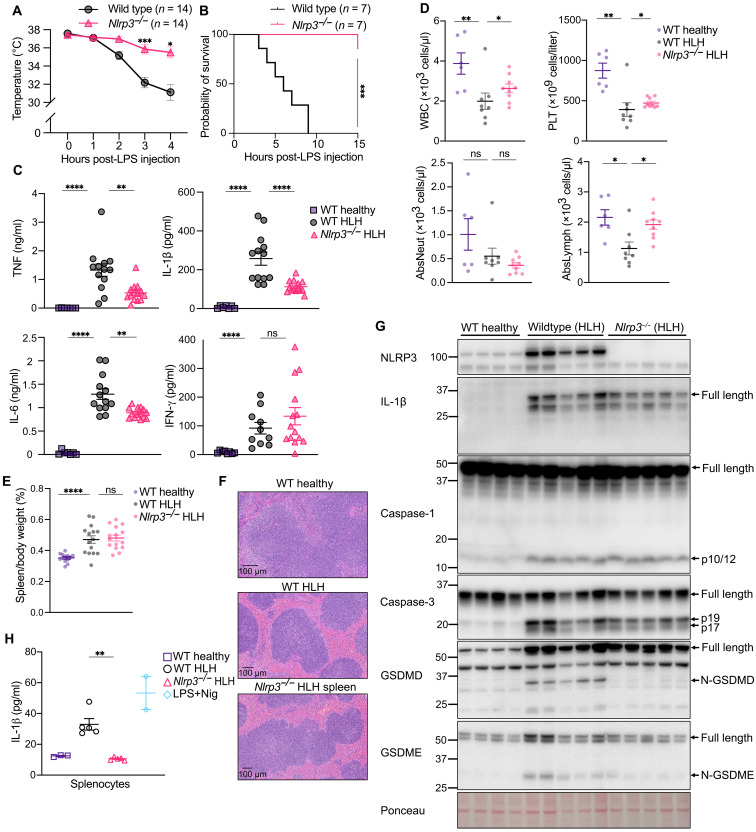
NLRP3 is a mediator of sHLH disease severity and increased cytokine levels. (**A**) Temperature of wild-type (WT) and *Nlrp3^−/−^* mice following sHLH induction. Pooled from three independent experiments, two that reached the ethical endpoint after 3 hours and one after 4 hours. (**B**) sHLH survival curve in WT and *Nlrp3^−/−^* mice. (**C**) Serum concentrations of IL-1β, IL-6, TNF, and IFN-γ in WT healthy (*n* = 6 to 7), WT HLH (*n* = 10 to 13), and *Nlrp3^−/−^* HLH (*n* = 14) mice 3 to 4 hours post-sHLH induction. ns, not significant. (**D**) Numbers of white blood cells (WBCs), platelets (PLTs), neutrophils, and lymphocytes in WT healthy (*n* = 6), WT HLH (*n* = 8), and *Nlrp3^−/−^* HLH (*n* = 9) mice 3 to 4 hours post-sHLH induction. (**E**) Spleen weights of WT healthy (*n* = 12), WT HLH (*n* = 15), and *Nlrp3^−/−^* HLH (*n* = 15) mice 3 to 4 hours after sHLH induction. (**F**) H&E staining of spleen tissue harvested from WT healthy, WT HLH, and *Nlrp3^−/−^* HLH mice 3 to 4 hours after sHLH induction (*n* = 5; see fig. S2 and table S1 for additional analysis). (**G**) Immunoblots of spleen tissue from WT healthy control mice (*n* = 4) or WT HLH (*n* = 5) and *Nlrp3^−/−^* HLH (*n* = 5) mice harvested 3 to 4 hours after sHLH induction. (**H**) IL-1β concentrations in the splenocyte supernatant from WT (*n* = 3), WT HLH (*n* = 5), and *Nlrp3^−/−^* HLH (*n* = 5) mice 24 hours after ex vivo culture. Splenocyte samples from healthy WT mice were treated with LPS (100 ng/ml) for 3 hours followed by nigericin (10 μM) for 40 min as a control. Data represent the means ± SEM. *P ≤ 0.05, **P ≤ 0.01, ***P ≤ 0.001, and ****P ≤ 0.0001. Mouse temperatures were analyzed using a two-way ANOVA, with a Bonferroni correction for multiple comparisons. An unpaired nonparametric Mann-Whitney test was used for analyzing cytokines, blood counts, and spleen weights. Survival graphs were analyzed using a log-rank (Mantel-Cox) test.

We adopted a mouse sHLH model that recapitulates core parameters of human sHLH, including elevated inflammatory cytokines, hyperferritinemia, hypertriglyceridemia, pancytopenia, hemophagocytosis, and highly lethal nature (*36*). Like human sHLH, this model is also triggered by pathogen ligands, requires the sequential injection of polyinosinic:polycytidylic acid (poly I:C; 10 mg/kg) for 24 hours, followed by low-dose lipopolysaccharide (LPS; 5 mg/kg), and is not caused by either agent alone or when they are administered in the reverse order (*36*). Consistent with previous studies using body temperature changes as a measure of sHLH disease severity, including lethality (*36*, *37*), wild-type (WT) control animals displayed a rapid body temperature drop following LPS administration (Fig. 1A) and, from ~4 hours, had to be euthanized upon reaching our ethical endpoint (moribund physical appearance and/or a temperature of ≤30°C) (Fig. 1B). The combined loss of caspase-1 and caspase-11 had no impact on the rate of body temperature loss (fig. S1), consistent with previous findings showing that lethality caused by this sHLH model occurs via Toll-like receptor 2 (TLR2)/TLR4 but independent of caspase-1 and caspase-11 (*36*). Therefore, poly I:C– and LPS-induced sHLH is distinct to the caspase-11–dependent sepsis model driven by high-dose LPS (54 mg/kg) administration, where large quantities of LPS allow for spontaneous macrophage uptake to trigger lethal noncanonical caspase-11 inflammasome signaling (*38*, *39*).

We hypothesized that sHLH may involve the inflammasome sensor protein NLRP3 as, despite the lack of involvement of caspase-1 and caspase-11 in this sHLH model, in certain contexts, TLR4 signaling activates the NLRP3 inflammasome (*40*–*42*) to engage apoptotic caspases (*28*, *43*–*47*). We found that NLRP3-deficient mice were significantly protected from sHLH, as measured by reduced core body temperature loss (Fig. 1A) and their increased probability of survival (Fig. 1B). In addition, elevated serum inflammatory cytokine levels that are associated with sHLH, including TNF, IL-1β, and IL-6, were all reduced in *Nlrp3^−/−^* mice when compared to wild-type control animals, although increased IFN-γ was not affected by NLRP3 deletion (Fig. 1C). The loss of NLRP3 also protected from sHLH-driven leukopenia, thrombocytopenia, and reduced lymphocytes but did not affect neutrophil numbers (Fig. 1D).

Splenomegaly is a common feature of sHLH, is one of the diagnostic criteria, and plays important roles in immune cell activation and cytokine production. The increased spleen-to-body weight ratio resulting from sHLH (Fig. 1E) and prominent red pulp congestion and apoptotic debris (Fig. 1F, fig. S2, A and B, and table S1) was not noticeably different between wild-type and *Nlrp3^−/−^* mice. Erythrophagocytosis was also detected in wild-type and *Nlrp3^−/−^* animals (fig. S2B), although it was not overly conspicuous.

Immunoblot analysis of spleens from healthy control and sHLH mice identified that sHLH resulted in an increase in both NLRP3 and precursor IL-1β levels (Fig. 1G). Although we could not detect processed (activated) IL-1β in spleen tissue lysates (Fig. 1G) and cultured splenocyte supernatants (fig. S2C), likely reflecting Western blot sensitivity limitations, we observed that spontaneous IL-1β release was increased in splenocytes isolated from sHLH mice, and this increase was abolished in splenocytes isolated from *Nlrp3^−/−^* sHLH animals (Fig. 1H). In addition, we detected significant levels of caspase-1 and caspase-3 processing (Fig. 1G), along with the cleavage of GSDMD and GSDME to their N-terminal membrane–associated pore-forming fragments (Fig. 1G). Notably, NLRP3 deficiency prevented GSDMD activation and mildly reduced GSDME activation in spleen tissue (Fig. 1G). Despite this, the cleavage of caspase-1 and caspase-3, which process GSDMD and GSDME, respectively, was not greatly altered between wild-type and *Nlrp3^−/−^* animals (Fig. 1G). We hypothesized that this discrepancy may reflect the broader expression of caspase-3 and caspase-1 in spleen tissue relative to NLRP3, GSDMD, and GSDME, thereby masking the ability to detect NLRP3-specific caspase-1 or caspase-3 activation. Consistent with this idea, caspase-3, cleaved caspase-3, and caspase-1 staining was extensively distributed in sHLH spleen tissue, while the distribution of GSDMD correlated with F4/80–positive macrophages (fig. S3A). Although we lacked suitable NLRP3 and GSDME antibodies compatible with immunohistochemistry, when combined with findings showing that increased sHLH serum IL-1β is mostly NLRP3 and caspase-1 dependent (Fig. 1C and fig. S1), these data suggest that apoptotic caspase-3 and NLRP3-independent caspase-1 activity occurs extensively throughout the spleen, while NLRP3-mediated activation of IL-1β, GSDMD, and GSDME is restricted to splenic phagocytes (fig. S3B).

### Combined deletion of GSDMD and GSDME, but not IL-1β and IL-18, confers protection from sHLH

**Fig. 2.**
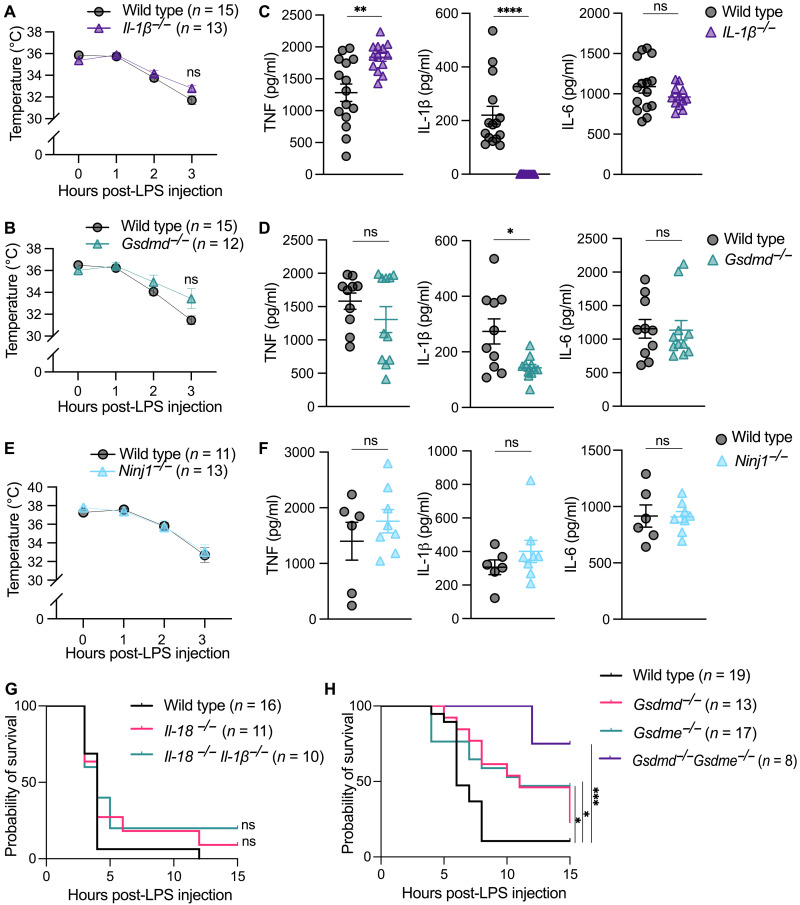
Co-deletion of GSDMD and GSDME protects from sHLH lethality. (**A** and **B**) Rectal temperatures of (A) WT (n = 15) and *Il-1*β*^−/−^* (*n* = 13) mice or (B) WT (*n* = 15) and *Gsdmd^−/−^* (*n* = 12) mice post-sHLH induction. Data are pooled from two (A) or three (B) independent experiments. (**C** and **D**) Serum concentration of IL-1β, IL-6, and TNF in (C) WT (*n* = 15) and *IL-1*β*^−/−^* (*n* = 13) mice or (D) WT (*n* = 10) and *Gsdmd^−/−^* (*n* = 11) mice 3 hours post-sHLH induction. (**E**) Rectal temperatures of WT (*n* = 11) and *Ninj1^−/−^* (*n* = 13) mice post-sHLH induction. Data are pooled from two independent experiments. (**F**) Serum concentration of IL-1β, IL-6, and TNF in WT (*n* = 6) and *Ninj1^−/−^* (*n* = 8) mice 3 hours post-sHLH induction. (**G** and **H**) Survival curve of (G) WT (*n* = 16), *Il-18^−/−^* (*n* = 11), and *Il-18^−/−^Il-1*β*^−/−^* (*n* = 10) mice or (H) WT (*n* = 19), *Gsdmd^−/−^* (*n* = 13), *Gsdme^−/−^* (*n* = 17), and *Gsdmd^−/−^ Gsdme^−/−^* (*n* = 8) mice post-sHLH induction. Data are pooled from either two (G) or three (H) independent experiments. (A to D) Data represent the mean values ± SEM. *P ≤ 0.05, **P ≤ 0.01, ***P ≤ 0.001, and ****P ≤ 0.0001. Mouse temperatures were analyzed using a two-way ANOVA, with a Bonferroni post hoc correction for multiple comparisons, while an unpaired nonparametric Mann-Whitney test was used for analyzing cytokine levels between genotypes. Survival graphs were analyzed using a log-rank (Mantel-Cox) test.

We next asked whether GSDMD and GSDME, ninjurin-1 (NINJ1)–driven cell lysis, and/or the pyroptotic-associated inflammatory cytokines IL-1β and IL-18 contributed to sHLH disease severity. The genetic removal of IL-1β or GSDMD did not significantly alter the rapid drop in temperature resulting from sHLH disease induction (Fig. 2, A and B), although *Gsdmd^−/−^* mice tended to display a nonsignificant minor protection from temperature loss at 3 hours (Fig. 2B). Furthermore, in contrast to NLRP3-deficient animals that had reduced levels of TNF, IL-1β, and IL-6 upon sHLH induction (Fig. 1C), but akin to caspase-1 and caspase-11 loss (fig. S1), mice lacking IL-1β or GSDMD only reduced serum IL-1β, not sHLH-driven TNF and IL-6 production (Fig. 2, C and D). Similarly, neither sHLH-mediated temperature loss (Fig. 2E) nor serum cytokine production (Fig. 2F) was affected by deletion of NINJ1, which drives cell lysis and the release of large-molecular-weight molecules downstream of gasdermin pore formation (*48*).

The above data suggest the potential for redundancy in either inflammasome-activated cytokines, such as IL-1β and IL-18, or cell death effectors, such GSDMD and GSDME, in causing sHLH lethality. We therefore generated mice lacking both IL-1β and IL-18. However, similar to IL-1β deficiency (Fig. 2A), the combined loss of these cytokines failed to improve sHLH survival rates or limit decreases in core body temperatures (Fig. 2G and fig. S4, A and B). In contrast, while GSDMD or GSDME deficiency modestly afforded protection from sHLH, mice deleted for both the pyroptotic effectors, GSDMD and GSDME, were significantly protected from reduced body core temperatures and showed enhanced survival (Fig. 2H and fig. S4, C and D). However, it is worth noting that we observed some variability across multiple independent experiments with regard to the morbidity-associated endpoint (fig. S4D). These data indicate that NLRP3 drives sHLH disease severity, at least in part, through redundancy in pyroptotic GSDMD- and GSDME-mediated cell death signaling.

### Caspase-8 and MLKL deficiency alleviates sHLH disease severity

NLRP3 inflammasome complexes can recruit caspase-8 that can drive caspase-3 activation of GSDME (*49*), which may help explain the strong lack of protection from sHLH in caspase-1–deficient mice (*36*). However, caspase-8 deletion can only be studied on a receptor-interacting serine/threonine-protein kinase 3 (RIPK3)– or mixed lineage kinase domain–like pseudokinase (MLKL)–deficient background, as otherwise the loss of caspase-8 ignites embryonically lethal RIPK3 and MLKL necroptotic signaling (*50*–*52*). Although we previously showed that combined RIPK3 and caspase-8 deletion protected from sHLH, RIPK3 deletion alone also reduced key HLH-associated cytokines in this model, such as IL-1β (*37*). We therefore examined sHLH severity in *Casp8^−/−^Mlkl^−/−^* and *Mlkl*^−/−^ mice as, unlike RIPK3, MLKL does not harbor diverse non-necroptotic signaling functions (*23*).

**Fig. 3.**
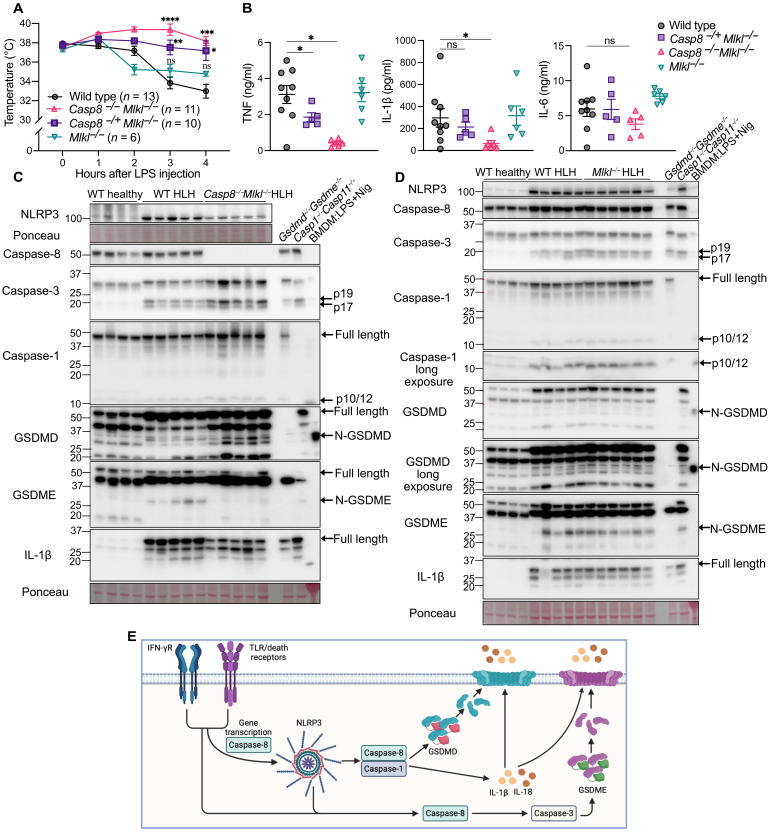
Caspase-8 and MLKL deletion protects from sHLH severity and increased inflammatory cytokines. (**A**) Temperatures of WT, *Casp8^−/−^Mlkl^−/−^*, *Casp8^−/+^Mlkl^−/−^*, and *Mlkl^−/−^* mice following sHLH induction. Pooled from two independent experiments involving WT, *Casp8^−/−^Mlkl^−/−^*, and *Casp8^−/+^Mlkl^−/−^* mice (one that reached the ethical endpoint after 3 hours and one after 4 hours) and one experiment using WT and *Mlkl^−/−^* animals. (**B**) Serum cytokine concentrations in WT (*n* = 9), *Casp8^−/−^Mlkl^−/−^* (*n* = 5 to 6), *Casp8^−/+^Mlkl^−/−^* (*n* = 5), and *Mlkl^−/−^* (*n* = 6) mice 3 to 4 hours post-sHLH induction. (**C** and **D**) Immunoblot analysis of spleen tissue from (C) WT healthy control (*n* = 4), WT HLH (*n* = 5), and *Casp8^−/−^Mlkl^−/−^* (*n* = 5) mice and (D) WT healthy control (*n* = 4), WT HLH (*n* = 5), and *Mlkl^−/−^* (*n* = 6) mice harvested 3 to 4 hours after sHLH disease induction. *Gsdmd^−/−^ Gsdme^−/−^* HLH spleen, *Casp1^−/−^Casp11^−/−^* HLH spleen, and BMDMs treated with LPS (100 ng/ml) followed by nigericin (10 μM) were used as controls. (**E**) Model of cell death signaling in sHLH based on this study and other published research. IFN-γ and/or TLR/death receptor signaling activates caspase-8 transcription for optimal inflammasome priming. NLRP3 recruitment of caspase-1 and caspase-8 via ASC allows for GSDMD cleavage and, in part, caspase-3 activation of GSDME (also engaged directly downstream of TLRs/death receptors). Gasdermin pores cause pyroptosis to drive disease severity, and although they facilitate the release of NLRP3-activated IL-1β and IL-18, ultimately, gasdermin-mediated release of these two cytokines alone does not account for the full spectrum of disease protection resulting from GSDMD and GSDME deletion. Data in (A) and (B) represent the mean values ± SEM. *P ≤ 0.05, **P ≤ 0.01, ***P ≤ 0.001, and ****P ≤ 0.0001. Temperatures were analyzed using a two-way ANOVA, with Tukey’s correction for multiple comparisons. An unpaired nonparametric Mann-Whitney test was used for analyzing cytokine levels. (E) created in BioRender. J. Vince (2025) https://BioRender.com/zhwmsiu.

Both *Casp8^−/−^Mlkl^−/−^* and *Casp8^+/−^Mlkl^−/−^* mice were protected from sHLH-induced temperature loss compared to wild-type and *Mlkl*^−/−^ animals, with the loss of both caspase-8 alleles affording the strongest protection (Fig. 3A). This gene dosage effect was mirrored by the levels of serum cytokines, with caspase-8 loss, but not MLKL, limiting sHLH-induced increases in IL-1β and TNF, but not IL-6, levels (Fig. 3B).

The reduced serum cytokines in *Casp8^−/−^Mlkl^−/−^* mice relative to wild-type and *Mlkl*^−/−^ animals may reflect the role of caspase-8 in driving cell death, gene transcription, or both (*49*). To address these possibilities, we analyzed spleen lysates by immunoblotting. This revealed that increased NLRP3 and IL-1β precursor levels in sHLH spleen tissue were lower, but not abolished, in *Casp8^−/−^Mlkl^−/−^* mice compared to wild-type and *Mlkl^−/−^* mice (Fig. 3, C and D). This finding likely reflects previous observations showing that macrophage caspase-8 is required for efficient NLRP3 and IL-1β transcription, termed inflammasome priming, in response to TLR ligand stimulation (*53*, *54*). On the other hand, caspase-8 also affected pyroptotic death signaling as its loss mitigated sHLH-induced activation of GSDME, but not GSDMD (Fig. 3, C and D). Why sHLH activation of GSDMD processing was not altered by the reduced NLRP3 levels observed in caspase-8 gene–targeted mice may reflect sufficient NLRP3 to still engage caspase-1, the major NLRP3-activated protease that cleaves GSDMD. Regardless, akin to NLRP3 deletion, active caspase-1 and caspase-3 also remained unchanged in the sHLH spleen tissue of *Casp8^−/−^Mlkl^−/−^* mice when compared to wild-type mice (Fig. 3C), supporting the idea of widespread NLRP3-independent caspase-1 and caspase-3 expression and activation (fig. S3).

Together, these data support a model where caspase-8 contributes to sHLH-like disease severity by regulating gene transcription (e.g., NLRP3 and inflammatory cytokines) and posttranslationally by promoting the cleavage of GSDME (and possibly GSDMD, redundantly with caspase-1), thereby playing a role both upstream and downstream of NLRP3 signaling (Fig. 3E).

### Small-molecule BET inhibitors can block NLRP3 inflammasome–dependent cell death

**Fig. 4.**
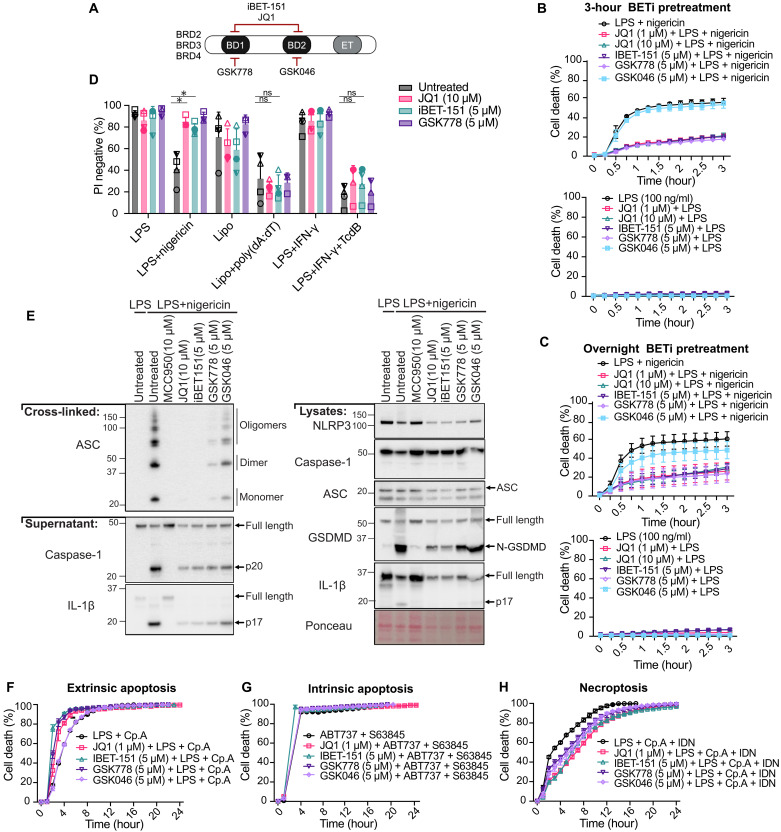
BETis limit NLRP3-mediated pyroptosis in macrophages. (**A**) BET family proteins and their targeting by BETis. (**B**) BMDMs were treated with JQ1, iBET-151, GSK778, and GSK046 for 3 hours before LPS (100 ng/ml) priming (3 hours) and nigericin (10 μM) treatment, and cell death was assessed. Data are pooled from two independent experiments. (**C**) BMDM death was assessed as in (B), except that BETis were given overnight before LPS and nigericin treatment. Data are pooled from four independent experiments. (**D**) BMDMs were treated with JQ1, iBET-151, and GSK 778 overnight followed by LPS (100 ng/ml) for 3 hours ± nigericin (10 μM) for 40 min, Lipofectamine (Lipo) ± poly(dA:dT) (1.5 μg/ml) for 4 hours, and LPS (200 ng/ml) + IFN-γ (50 ng/ml) for 3 hours ± TcdB toxin (1 μg/ml) for 6 hours. Cell death was assessed by flow cytometry. Data are pooled from four independent experiments. (**E**) Immunoblot of BMDMs treated with BETi overnight and then with LPS (100 ng/ml) for 3 hours followed by nigericin (10 μM) for 30 min. Representative of three independent experiments. (**F** to **H**) BMDMs were treated with BETi overnight and then stimulated with (F) LPS (100 ng/ml) and Cp.A (1 μM), (G) ABT737 (1 μM) and S63845 (10 μM), or (H) LPS (100 ng/ml), Cp.A (1 μM), and IDN-6556 (IDN, 10 μM), and cell death was assessed. A second independent experiment is shown in fig. S5 (C to E). (B to D) Data represent the mean values ± SEM pooled from the indicated number of experiments. *P ≤ 0.05. An unpaired nonparametric Mann-Whitney test was used for cell death responses (D). (A) created in BioRender. J. Vince (2025) https://BioRender.com/ta9lewc.

Current sHLH treatments, while effective in some cases, are not universally successful. We therefore sought to identify new inhibitors of NLRP3 or pyroptotic signaling that might be applied to limit sHLH disease severity. Small-molecule BETi targeting of the epigenetic reader BRD–containing proteins BRD2, BRD3, and BRD4 (Fig. 4A) has been developed to cause cancer cell death (*55*, *56*). However, BETis have also emerged as potential anti-inflammatory compounds because of their capacity to block cytokine gene transcription (*57*, *58*). While screening the capacity of BETis to affect programmed cell death in macrophages by IncuCyte live cell imaging of propidium iodide (PI) uptake, we found that both short (3 hours) and extended (overnight) BETi pretreatment inhibited LPS- and nigericin-mediated, NLRP3-driven pyroptosis (Fig. 4, B and C) while not affecting untreated (fig. S5A) or LPS-treated (Fig. 4, B and C) macrophage viability. Pan-BETis (i.e., JQ1 and iBET-151) and BETis selectively targeting bromodomain 1 (BD1) of BRD2/3/4 (GSK778; Fig. 4A) (*55*, *59*, *60*) blocked NLRP3 killing equivalently, while the BETi selective for the BD2 of BRD2/3/4 (GSK046) (*60*) did not efficiently prevent NLRP3-driven pyroptosis (Fig. 4, A to C). Flow cytometry and PI staining recapitulated these findings, showing that the loss of macrophage viability following nigericin treatment was largely rescued by pan and BD1-selective BETi treatment (Fig. 4D and fig. S5B). On the other hand, macrophage treatment with BETis did not alter pyroptosis caused by poly(deoxyadenylic-thymidylic) acid [poly(dA:dT)] transfection and activation of the AIM2 inflammasome or *Clostridium difficile* toxin B (TcdB) activation of the Pyrin inflammasome (Fig. 4D), suggesting that BETi targeting was specific for NLRP3.

When triggered, NLRP3 binds and nucleates oligomerization of the inflammasome adaptor protein ASC, which can recruit and activate caspase-1 to cleave GSDMD and IL-1β. Like the NLRP3 inhibitor MCC950, BD1-binding BETis (i.e., JQ1, iBET-151, and GSK778) all reduced ASC oligomerization in macrophages following nigericin treatment (Fig. 4E) and consequently limited caspase-1, GSDMD, and IL-1β processing into their bioactive fragments, including the release of cleaved caspase-1 and IL-1β into the cell supernatant (Fig. 4E). In contrast, BETis did not prevent extrinsic apoptosis (LPS and Smac-mimetic compound A (Cp.A) treatment; Fig. 4F and fig. S5C), intrinsic apoptosis (BH3-mimetic ABT737 and S63845 treatment; Fig. 4G and fig. S5D), or necroptosis (LPS, Cp.A, and pan-caspase IDN-6556 inhibition; Fig. 4H and fig. S5E). Therefore, BETis originally identified as inducers of cancer cell death (*55*) act unexpectedly to block NLRP3-driven pyroptosis and, at least in macrophages, are relatively specific to this particular cell death pathway.

### BETis limit inflammasome priming to block NLRP3 activity

**Fig. 5.**
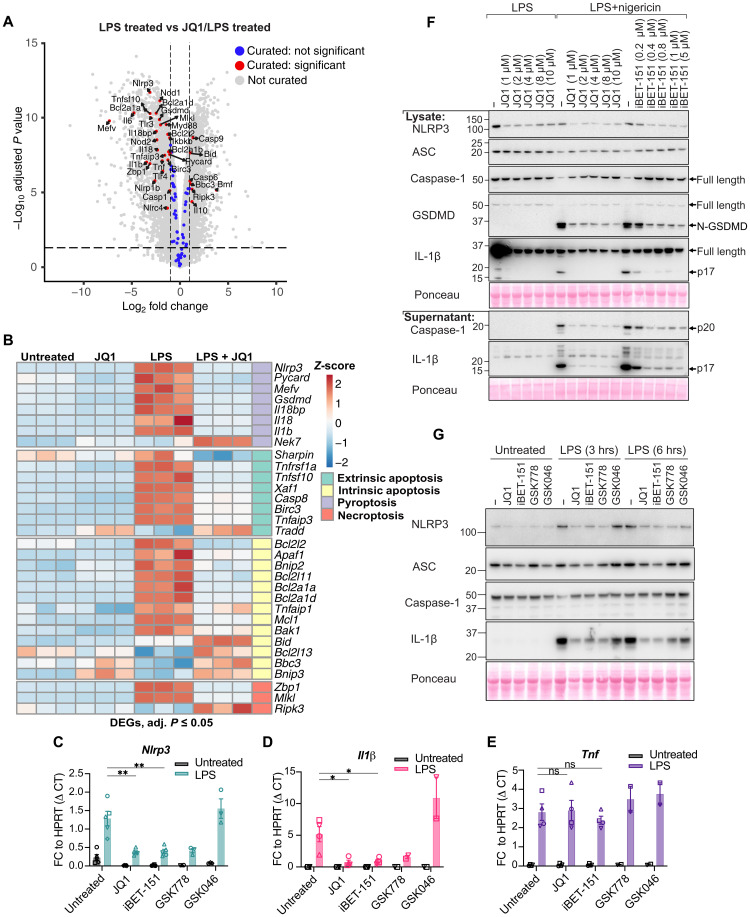
BETis reduce the expression of NLRP3 and IL-1β at the transcriptional level and block pyroptosis. (**A**) Volcano plot displaying DEGs associated with different cell death signaling pathways enriched in BMDMs treated with ±JQ1 (0.5 μM) for 12 hours followed by ±LPS (50 ng/ml) for 4 hours. Adjusted *P* value <0.05 and cutoff values of logFC ≥ 1 or logFC ≤ −1 (*n* = 3 biological replicates). Data are reanalyzed from M. Hoffner O’Connor *et al*. (*62*). (**B**) Heatmap of DEGs associated with different cell death signaling pathways in BMDMs treated with ±JQ1 (0.5 μM) for 12 hours followed by ±LPS (50 ng/ml) for 4 hours. Adjusted *P* value <0.05 and cutoff values of logFC ≥ 1 or logFC ≤ −1 (*n* = 3 biological replicates). Data are re-analyzed from M. Hoffner O’Connor *et al*. (*62*). (**C** to **E**) BMDMs were treated with JQ1, iBET-151, GSK778, and GSK046 overnight and then with LPS (100 ng/ml) for 3 hours. *Nlrp3*, *Il1*β, and *Tnf* expression was measured by qPCR using *Hprt* as the housekeeping gene. Each symbol represents a biological replicate. Data are pooled from two to five independent experiments. (**F**) Immunoblot of BMDMs that were treated with JQ1 and iBET-151 overnight and then with LPS (100 ng/ml) for 3 hours followed by nigericin (10 μM) for 30 min (representative of two independent experiments). (**G**) Immunoblot of BMDMs treated with JQ1 (10 μM), iBET-151 (5 μM), GSK778 (5 μM), and GSK046 (5 μM) overnight and then stimulated with LPS (100 ng/ml) for 3 and 6 hours (representative of three independent experiments). hrs, hours. Data (C to E) represent the mean values ± SEM from the indicated number of independent experiments. *P ≤ 0.05 and **P ≤ 0.01. An unpaired nonparametric Mann-Whitney test was used for analyzing qPCR data.

We next investigated how BETis might limit NLRP3 inflammasome activity. Given the prominent role of BET proteins in regulating chromatin remodeling to promote gene transcription (*61*), we hypothesized that BETis prevent NLRP3 activity by reducing levels of NLRP3 transcript and, hence, the amount of NLRP3 protein available to be activated. First, we reanalyzed publicly available RNA sequencing data in which the same cell type, bone marrow–derived macrophages (BMDMs), was treated with the same BETi, JQ1, in a similar manner to our own experiments, JQ1 pretreatment followed by LPS stimulation (*62*). The analysis of genes involved in different cell death modalities (table S2) indicated that, as expected, LPS induced inflammasome priming with increased expression of NLRP3, IL-1β, and IL-18 (Fig. 5, A and B, and fig. S6). Notably, JQ1 pretreatment prevented the up-regulation of these genes and also reduced the expression of several transcripts involved in apoptotic and necroptotic signaling (Fig. 5, A and B). As JQ1 only affected macrophage killing to canonical NLRP3 activation and did not affect these distinct apoptotic and necroptotic cell death modalities (Fig. 4, F to H, and fig. S5, C to E), these results suggest that these alternate cell death pathway components are sufficiently stable at the protein level to maintain functional capacity.

To confirm the on-target specificity for JQ1 on reducing NLRP3 and IL-1β gene transcription, we performed quantitative polymerase chain reaction (qPCR) using our suite of BETis that block NLRP3-driven pyroptosis (JQ1, iBET-151, and GSK778) or BD2-targeting GSK046, which does not. This analysis confirmed that LPS induced NLRP3 transcription, and this was only reduced by BETis that prevent NLRP3-induced pyroptotic death (Fig. 5C). Similarly, these same BETis (i.e., JQ1, iBET-151, and GSK778) also prevented TLR-induced IL-1β transcription (Fig. 5D) but, unexpectedly, had less of an impact on LPS-induced TNF mRNA (Fig. 5E). These changes in transcript levels were validated at the protein level, where BETi treatment limited the LPS (TLR4)– and Pam3CSK4 (TLR1/2)–induced expression of NLRP3 and IL-1β (Fig. 5, F and G, and fig. S7A) in addition to IL-6 (fig. S7B). In comparison, BETis were more effective at reducing Pam3CSK4-induced TNF when compared to LPS (fig. S7B). These data indicate that BETis can reduce pro-inflammatory gene induction, particularly NLRP3-related genes, and that they display some selectivity in their ability to perturb signaling from distinct TLRs.

Despite JQ1 reducing *Mefv* (encodes for Pyrin), *Pycard* (encodes for ASC), and *Gsdmd* transcript levels to varying degrees (Fig. 5, A and B), it failed to greatly affect levels of constitutively expressed ASC and GSDMD proteins (Fig. 5, F and G, and fig. S7A). Consequently, JQ1 treatment did not alter pyroptotic macrophage death caused by the activation of other inflammasomes reliant on Pyrin, ASC, and GSDMD (Fig. 4D). These findings were recapitulated in the human monocytic THP-1 cell line, where BD1-targeting BETis (but not BRD2- or BD2-targeting BIC-1 and GSK046, respectively) were effective at shutting down NLRP3 and IL-1β protein expression, but not caspase-1, ASC, or GSDMD (fig. S7C).

BET proteins have been reported to regulate transcription factors, such as RelA (p65), required for *Nlrp3* expression, suggesting that they may indirectly promote *Nlrp3* gene transcription downstream of TLR stimulation (*63*). In support of this idea, JQ1 treatment reduced the mRNA transcript of several components required for efficient TLR signaling, including *IkB* kinases, adaptor components *Myd88* and *Tirap*, and *RelA* itself (fig. S8A). Consistent with these data, immunoblot analysis showed that BETi treatment reduced the efficiency of LPS-triggered p65 and JNK phosphorylation, which correlated with a small, yet reproducible, decrease in overall levels of p65 (fig. S8B). These findings align with research showing that only minor defects in transcriptional regulators can abrogate inflammatory protein production (*64*) and show, at least in part, that BETis act to block pyroptosis by reducing the ability of TLRs to signal correctly and prime the NLRP3 inflammasome.

### BETis limit sHLH severity and reduce NLRP3 levels in diseased tissue

**Fig. 6.**
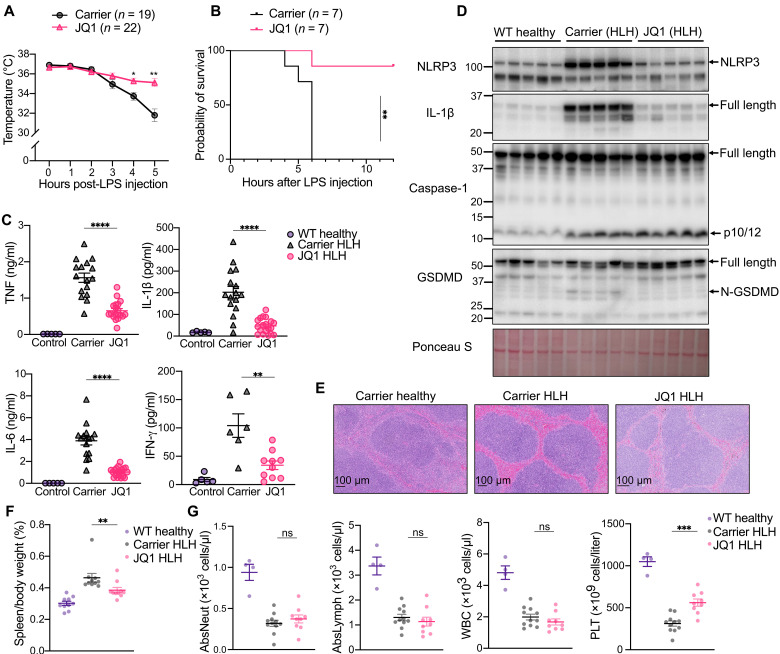
JQ1 protects from sHLH disease severity by reducing NLRP3 and inflammatory cytokine levels. (**A**) Rectal temperature of mice following sHLH induction treated with JQ1 (50 mg/kg) (*n* = 22) or vehicle (DMSO) (*n* = 19). Data are pooled from four independent experiments, two of which reached the ethical endpoint after 4 hours (carrier, *n* = 8; JQ1, *n* = 11) and another two after 5 hours (carrier, *n* = 11; JQ1, *n* = 11). (**B**) Survival curve following sHLH induction in carrier HLH (*n* = 7) and JQ1 HLH (*n* = 7) mice. (**C**) Serum/plasma concentrations of IL-1β, IL-6, TNF, and IFN-γ of mice treated in (A). (**D**) Immunoblot analysis of spleen tissue harvested from healthy mice (*n* = 5), upon sHLH induction in carrier-treated animals (*n* = 5), or in JQ1-treated mice (*n* = 5), as described in (A). (**E**) H&E staining of spleen tissue harvested from WT healthy, carrier HLH, and JQ1 HLH mice 3 to 4 hours after sHLH induction (*n* = 3 to 4). Additional mice are shown in fig. S9. (**F**) Spleen weight–to–body weight percentage of WT healthy (*n* = 10), carrier HLH (*n* = 10), and JQ1 HLH (*n* = 10) mice 3 to 4 hours after sHLH induction. (**G**) Blood counts showing the numbers of white blood cells, platelets, neutrophils, and lymphocytes in WT healthy (*n* = 4), carrier HLH (*n* = 11), and JQ1 HLH (*n* = 9) mice 3 to 4 hours post-sHLH induction. (A, C, F, and G) Data represent the mean values ± SEM. *P ≤ 0.05, **P ≤ 0.01, ***P ≤ 0.001, and ****P ≤ 0.0001. Mouse temperatures were analyzed using a two-way ANOVA, with a Bonferroni post hoc correction for multiple comparisons, while an unpaired nonparametric Mann-Whitney test was used for analyzing cytokine levels, blood counts, and spleen weight to body weight.

We reasoned that because NLRP3 loss protected from sHLH, then BETis may reduce NLRP3 levels in vivo, thereby providing a means to limit pathological NLRP3 inflammasome signaling. To investigate this, mice were treated intraperitoneally with the BETi JQ1 before inducing sHLH. Similar to NLRP3-deficient mice (Fig. 1), JQ1 treatment, but not the carrier control, significantly protected mice from sHLH-driven body temperature reductions (Fig. 6A), increased the probability of survival (Fig. 6B), and limited serum increases in IL-1β, TNF, and IL-6 (Fig. 6C). Moreover, analysis of spleen tissue demonstrated that increased sHLH-induced NLRP3 and IL-1β levels were blocked by JQ1 treatment, as was NLRP3-dependent GSDMD cleavage to its pore-forming N-terminal fragment (Fig. 6D). Also consistent with NLRP3 deletion (Fig. 1), sHLH-induced caspase-1 processing was not altered by JQ1 treatment (Fig. 6D), nor did JQ1 prevent the gross splenic histopathological features of sHLH (Fig. 6E, fig. S9, and table S3).

Despite JQ1 treatment reducing sHLH induction of NLRP3 and recapitulating the above features observed in NLRP3-deficient mice, JQ1 altered several parameters that reduced NLRP3 levels could not account for. Specifically, unlike NLRP3 loss, the administration of JQ1 significantly reduced sHLH-induced serum IFN-γ (Fig. 6C) and splenomegaly (Fig. 6F). However, JQ1 did not provide any rescue of sHLH-mediated leukopenia, lymphopenia, and neutropenia, although it did confer some protection from thrombocytopenia (Fig. 6G). Therefore, BETis likely limit sHLH disease severity via multiple mechanisms, including their ability to reduce NLRP3 expression and downstream pyroptotic signaling, as well as the production of inflammatory IL-1β, IL-6, TNF, and IFN-γ that contribute to the cytokine storm.

## DISCUSSION

Despite the increased use of targeted therapies to treat HLH, such as JAK (*65*), IL-1 (*66*), IL-6 (*67*, *68*), and IFN-γ inhibitors (*69*), with a 5-year survival rate of ~60%, there remains a clear need to define the immune cell signaling pathways that contribute to disease. This is particularly relevant for sHLH where, unlike primary HLH, disease pathogenesis is less clear because of the lack of defined genetic lesions. Here, we show that NLRP3 activation and combined GSDMD and GSDME signaling contribute to the pathogenesis of an sHLH mouse model. In addition, we identify that BD1-targeted BETis represent a cell death–targeted therapy that can block NLRP3 inflammasome pyroptotic responses in macrophages and that this mode of action likely limits sHLH disease severity.

Genetic deletion of NLRP3 protected from sHLH lethality and not only reduced levels of serum IL-1β, as expected, but also both TNF and IL-6, cytokines not produced via direct inflammasome engagement. This is reminiscent of liposomal RNA vaccines where NLRP3 inflammasome–driven IL-1β controls the production of several inflammatory mediators, including TNF and IL-6 (*70*). However, in the context of sHLH, IL-1β deletion did not reduce TNF and IL-6 levels, indicating that other immunogenic molecules released during pyroptosis are likely required to induce TNF and IL-6 production. These molecules must also be appropriately sized to traverse the ~22-nm gasdermin pore because, unlike GSDMD and GSDME loss, the deletion of NINJ1 that allows the release of large-molecular-weight proteins downstream of gasdermin activation had no impact on sHLH pathogenesis. Upstream of NLRP3 and gasdermin triggering, it is also noteworthy that the combined loss of death ligand signaling (TNF, FasL, and TRAIL) and dual TNF and IFN-γ inhibition is protective in the sHLH model we used in this study (*37*, *71*). As such, from a biological therapy viewpoint, sHLH would likely benefit most from combination anticytokine therapies or small molecules such as BETis that, as we show, limit sHLH-driven production of several inflammatory mediators, including IL-1β, IL-6, TNF, and IFN-γ, that contribute to the cytokine storm.

Whether pathological NLRP3 inflammasome activity contributes to disease pathology in primary HLH caused by distinct genetic defects or is hyperactive in other sHLH models and patient cells remains to be fully evaluated. For example, small-molecule NLRP3 and caspase-1 inhibitors showed no clinical benefit in a mouse model of sHLH resulting from repeated TLR9 stimulation despite reducing plasma IL-18 (*72*). However, the failure of these inhibitors to block caspase-1 processing in the diseased liver may indicate ineffective tissue penetration and efficacy (*72*), and in the absence of caspase-1 activity, caspase-8 may substitute to enable inflammasome responses (*45*, *46*, *73*, *74*); therefore, genetic studies in this alternate sHLH model would be of interest to pursue. Similarly, in a model of primary HLH induced in perforin-deficient mice by infection with lymphocytic or mouse choriomeningitis virus, the genetic removal of ASC or caspase-1 failed to prevent HLH lethality despite a large reduction in IL-18 levels (*75*). Although the genetic role of NLRP3 was not examined in this study, one would expect that it would mirror the loss of ASC, a key NLRP3 adaptor. Last, despite XIAP loss predisposing to primary HLH and heightened NLRP3 inflammasome engagement, recent studies suggest that considerable cell death signaling redundancy exists following pathogen ligand sensing in XIAP-deficient HLH models, with apoptotic caspase-8 capable of driving both GSDMD and IL-1β activation in the absence of NLRP3 (*6*, *40*, *41*). Therefore, the role of NLRP3 in HLH may be context specific and depend on several factors, such as the HLH trigger and relevant genetic lesions.

Our results show that caspase-8 deletion considerably limits sHLH severity in mice and is associated with reduced levels of NLRP3 and inflammatory cytokines such as TNF and IL-1β, as well as the loss of GSDME processing to its pore-forming fragment. The signaling pathways driving sHLH severity in the model we used, including TLR2/4 (*36*), death ligands (*37*), and IFN-γ (*71*), all have the capacity to activate caspase-8. This alone likely suffices for canonical caspase-8/caspase-3–mediated activation of GSDME. However, our data showing a partial reduction in GSDME cleavage in *Nlrp3^−/−^* mice align with studies indicating that caspase-8 can also bind the inflammasome adaptor protein ASC to be recruited into NLRP3-ASC complexes and, via subsequent caspase-3 engagement, trigger GSDME (*24*, *44*–*46*, *76*, *77*). While, in certain contexts, caspase-8 can signal NLRP3 activation in both mouse (*41*) and human innate immune cells (*42*), the loss of caspase-8 did not prevent sHLH-induced, NLRP3-dependent GSDMD activation, arguing that caspase-8 deficiency does not abrogate NLRP3 activity in this context. Overall, these findings align with the idea that caspase-8 can act both upstream (e.g., via efficient inflammasome priming) and downstream (e.g., via processing of GSDME) of NLRP3 engagement.

The redundancy built into NLRP3 signaling was recently illustrated by research showing that, in the absence of GSDMD, GSDME can step in to induce cell death and IL-18 and IL-1β release in gain-of-function NLRP3 mutant mice (*78*). Our study complements and extends these findings showing that, in sHLH, wild-type NLRP3 also likely has the potential to engage both GSDMD and GSDME and that, when compared to the loss of a single gasdermin, the combined deletion of GSDMD and GSDME confers a greater survival advantage. The partial protection from lethal sHLH resulting from GSDMD deficiency alone aligns with reports that GSDMD loss reduces systemic inflammation in the CpG-TLR9–driven HLH model (*79*). However, as our data demonstrate that combined IL-1β and IL-18 removal does not mirror the protection afforded by GSDMD and GSDME co-deletion, then the release of other inflammatory cytokines, such as IL-1α, or molecules, such as a tissue factor (*80*), may contribute to the cytokine storm and lethal sHLH. Notably, studies have demonstrated that macrophage depletion improves survival in sHLH, whereas neutrophil depletion worsens it, highlighting that macrophages are key drivers of pathology (*81*, *82*).

Our identification of BETis as relatively specific blockers of NLRP3 inflammasome–driven pyroptosis and protection from sHLH is surprising on two fronts. First, this class of compounds was previously shown to trigger cell death signaling in transformed cells, not inhibit it (*55*, *83*). Second, despite modulating mRNA transcript levels of other inflammasome components, as well as the machinery involved in apoptotic and necroptotic cell death modalities, this was insufficient to significantly affect the execution of these alternative cell death pathways in macrophages. This may reflect the stability of relevant proteins in these pathways or the counterbalancing of reduction in both prosurvival mRNA transcripts (e.g., *Mcl-1*) and proapoptotic transcripts (e.g., *Bak*). Regardless, the specificity of BD1-targeted BETis for blocking NLRP3-driven pyroptosis and production of the potent inflammatory mediators such as IL-1β, TNF, and IFN-γ make these compounds interesting candidates to limit sHLH-driven cell death and cytokine storms. As such, antibody BETi conjugates to drive selective innate immune cell uptake may offer a targeted sHLH approach and would help prevent the dose-limiting systemic toxicities that have been identified from BETi clinical trials.

## MATERIALS AND METHODS

### Mice

The *Ninj1^−/−^* mice were generated by the Melbourne Advanced Gene Editing Centre (MAGEC) laboratory at the Walter and Eliza Hall Institute of Medical Research (WEHI) on a C57BL/6J background. Specifically, to make *Ninj1^fl/fl^* and *Ninj1^−/−^* mice, Cas9 mRNA (20 ng/μl), of single guide RNAs (10 ng/μl; CACACACTGGTCTCTAGCGG and TGTCGACAGCTGGAGTAATA), and a targeting vector to introduce the *Ninj1 flox* allele (sequence available upon request) were injected into the pronucleus of fertilized one-cell stage embryos generated from wild-type C57BL6/J breeders. After 24 hours, two-cell stage embryos were transferred into the uteri of pseudo-pregnant female mice. Viable offspring were genotyped by next-generation sequencing. *Nlrp3^−/−^* (*84*), *Gsdmd^−/−^* (*19*), *Gsdme^−/−^* [provided by Genentech (*48*)], *Gsdmd^−/−^Gsdme^−/−^* (generated in-house), *Casp1^−/−^Casp11^−/−^* (*85*), *Il1*β*^−/−^* (*86*), *Il1*β*^−/−^Il18^−/−^* (generated in-house), *Casp8^−/−^Mlkl^−/−^* (*50*), and *Mlkl^−/−^* (*87*) mice were all derived on, or backcrossed onto, a C57BL/6 background and were housed under specific pathogen–free conditions at WEHI. All procedures received approval from the WEHI Animal Ethics Committee (2020.038). All mice used in experiments had not been involved in prior procedures. The animals were in good health and were selected without regard to gender to generate BMDMs. Both female and male mice were at least 8 weeks old at the time of experimentation.

### Bone marrow–derived macrophages

Bone marrow was harvested from femoral and tibial bones of mice. Cells were cultured at 37°C in 10% CO_2_ on 15-cm bacterial petri dishes in 15 ml of Dulbecco’s modified Eagle’s medium containing 10% fetal bovine serum (FBS; Sigma-Aldrich or Bovogen), penicillin (50 U/ml), and streptomycin (50 mg/ml) and supplemented with 20% L929 cell–conditioned medium. Another 10 ml of culture media was added to cells on day 3. On day 6, differentiated macrophages were harvested and counted, and 5 × 10^5^ cells per well were plated into 24-well tissue culture–treated plates for immunoblot experiments or in 24-well non–tissue culture–treated plates for flow cytometry analysis. For IncuCyte live cell imaging, 5 × 10^4^ cells were plated per well into 96-well tissue culture–treated plates.

### Human monocytic THP-1 cells

THP-1 monocytic cells (TIB-202) were cultured in RPMI 1640 (Life Technologies) containing 10% FBS (Sigma-Aldrich), penicillin (100 U/ml), and streptomycin (0.1 mg/ml) at 37°C in 5% CO_2_. For experiments, 4 × 10^5^ cells were plated per well into 24-well tissue culture–treated plates.

### sHLH model

sHLH-like disease was induced in mice by sequential injection of poly I:C and LPS, as previously described (*36*). Mice received intraperitoneal injections of high-molecular-weight poly I:C (InvivoGen, tlrl-pic) in phosphate-buffered saline (PBS) at a 10 mg/kg body weight reconstituted in sterile endotoxin-free water and diluted with 5× PBS. After 24 hours, this injection was followed by intraperitoneal injection of LPS-B5 (InvivoGen, tlrl-pb5lps) at a 5 mg/kg body weight reconstituted in sterile endotoxin-free water and diluted with 5× PBS. Healthy control mice were intraperitoneally injected with PBS only. In JQ1-treated cohorts, mice received intraperitoneal injections of JQ1 (MedChemExpress, HY-13030) at a 50 mg/kg body weight reconstituted in dimethyl sulfoxide (DMSO) and diluted in the 10% carrier (2-hydroxypropyl)-β-cyclodextrin (MedChemExpress, HY-101103) 3 hours before poly I:C and LPS injections. Control mice received equivalent doses of DMSO and carrier. Core body temperatures were measured using a rectal thermometer (Oakton Instruments, WD-85000-00) before the LPS injection and every hour thereafter until the ethical endpoint was reached (moribund physical appearance and/or a temperature of ≤30°C). Mice were euthanized, and cardiac bleeds and organ harvesting were performed. In survival experiments, each mouse was euthanized once the ethical point was reached or after 15 hours if it was still healthy. Experiments used age-matched 8- to 14-week-old mice of both sexes as no gender-based differences in sHLH disease were observed. Animal studies were conducted in accordance with the GSK Policy on the Care, Welfare, and Treatment of Laboratory Animals and were approved by the Institutional Animal Ethics Committee at WEHI.

### Cell stimulations

BMDMs were pretreated with JQ1 (1 to 10 μM, Adoq Bioscience, A12729), iBET-151 (0.2 to 10 μM, MedChemExpress, HY-13235), GSK778 (5 to 10 μM, provided by GSK), GSK046 (5 to 10 μM, provided by GSK), and BIC-1 (5 μM, Merck, 203830) overnight followed by LPS (100 ng/ml, InvivoGen; tlrl-3pelps) or Pam3CSK (500 ng/ml, InvivoGen, vac-pms) treatment for 3 to 6 hours. For pyroptotic cell death analysis, BMDMs were pretreated with the indicated BETis for 3 hours or overnight. This was followed by LPS priming for 3 hours and then nigericin (10 μM, Sigma-Aldrich; N7143) treatment for 30 to 45 min. Other treatments to induce different cell death pathways were performed on BETi-pretreated macrophages using LPS (100 ng/ml) and the Smac-mimetic Cp.A (1 μM, TetraLogic Pharmaceuticals) to trigger extrinsic apoptosis, the BH3-mimetics ABT-737 (1 μM, Active Biochem; A-1002) and S63845 (10 μM, Active Biochem; A-6044) to induce intrinsic mitochondrial apoptosis, LPS (100 ng/ml) combined with Cp.A (1 μM) and the pan-caspase inhibitor IDN-6556 (10 μM, IDN, TetraLogic Pharmaceuticals) to cause necroptosis, Lipofectamine 2000 (Invitrogen, 11668-019) transfection of poly(dA:dT) (1.5 μg/ml, InvivoGen, tlrl-patn) to activate the AIM2 inflammasome, and LPS (200 ng/ml) and IFN-γ (50 ng/ml, recombinant mouse, R&D; 485-MI) cotreatment for 3 hours followed by TcdB protein (1 μg/ml, ab124001, Cambridge, UK) stimulation for 6 hours to engage the Pyrin inflammasome.

### ASC oligomerization

ASC oligomerization was assessed on the basis of the published protocol (*88*). Briefly, 2 × 10^6^ BMDMs were plated in six-well tissue culture–treated plates, treated with BETi overnight, and then primed with LPS for 2 to 3 hours. MCC950 (10 μM, provided by A. Roberson and M. Cooper, University of Queensland, Australia) was added to cells 10 min before LPS treatment. The culture medium was then replaced with Opti-MEM, and cells were stimulated with nigericin for 30 to 40 min. Plates were centrifuged at 1800*g* for 5 min at 4°C. Supernatants were collected, and cell pellets were washed with ice-cold PBS. After removing PBS, 225 μl of Milli-Q water containing an EDTA-free protease inhibitor (Roche) was added, followed by 25 μl of 10× PBS, resulting in a total volume of 250 μl. Cells were freeze thawed at 80°C and harvested. The cell lysate was centrifuged at 6000*g* for 20 min at 4°C. Supernatants were discarded, and the pellet was resuspended in 200 μl of ice-cold PBS. Disuccinimidyl suberate (Thermo Fisher Scientific, 21655) was added to a final concentration of 2 mM, and the samples were incubated at 37°C for 45 min with agitation every 5 to 10 min. After the incubation, the samples were centrifuged at 21,000*g* for 20 min at 4°C. Supernatants were removed, and the pellets were resuspended in Western blot sample buffer.

### Flow cytometry

BMDMs were detached using EDTA (5 mM) in PBS and added to the collected supernatant. PI (1 to 2 μg/ml, Sigma-Aldrich, P4170) was added to all samples, and PI-positive cells were quantified using an LSR II flow cytometer (Becton Dickinson, NJ). For each sample, PI exclusion analysis involved 10,000 nondebris single-cell events. Data analysis was performed using FlowJo version 9.

### IncuCyte imaging

BMDMs were plated on 96-well tissue culture–treated plates at 5 × 10^4^ cells per well and treated as indicated in the relevant figure legends. Imaging was performed by the IncuCyte S3 (Sartorius) system using SPY505-DNA (1:1000, Spirochrome, SC101) to stain all cells and PI (0.5 μg/ml) to stain dead cells. Bright-field, red florescent (acquisition time: 350 ms; PI-positive cells), and green florescent (acquisition time: 300 ms; all cells) images were acquired using a 96-well Corning Falcon (All Clr) vessel type and a 20× objective. In one experiment (fig. S2, C to E), SPY700-DNA (1:1000, SC601) was used to stain all cells, and images were taken using an IncuCyte SX5 (Sartorius). Analysis was carried out with Sartorius software, and the percentage of cell death was calculated by dividing the number of PI-positive cells by the total number of cells.

### Immunoblotting

Cells or cell-free supernatants were denatured in a 2% SDS lysis buffer containing 143 mM β-mercaptoethanol. Samples were resolved using 20-well 4 to 12% gradient gels (Invitrogen, WG1402BOX), and the protein was transferred onto Immoblon-E polyvinyl difluoride membranes (Merck Millipore; IEVH85R) or nitrocellulose (Amersham, 88018). Ponceau staining was performed to assess protein loading. Membranes were blocked in tris-buffered saline containing 5% skim milk (Devondale) and 0.1% Tween 20 (TBS-T) for 1 hour. After washing membranes with TBS-T, they were probed with primary antibodies overnight at 4°C (diluted in 5% bovine serum albumin or 5% skim milk in TBS-T at 1:1000). Primary antibodies used were as follows: NLRP3 (Adipogen, AG-20B-0014-C100), caspase-1 (Adipogen, AG-20B-0042-C100; Abcam, ab179515), GSDMD (Abcam, ab209845), IL-1β (R&D, AF-401-NA), cleaved caspase-3 (Cell Signaling, 9661), cleaved caspase-8 Asp^387^ (Cell Signaling, 9429 and 8592), pro-caspase-8 (in-house, 3B10), GSDME (Abcam, ab215191; Cell Signaling, 88874), MyD88 (Cell Signaling, 4283), NF-κB p65 (Cell Signaling, 6956), phospho-NF-κB p65 (Cell Signaling, 3033), phospho-p44/p42 MAPK (Cell Signaling, 9101), p38 (Cell Signaling, 9212), phospho-p38 (Cell Signaling, 4511), JNK (Cell Signaling, 9592), phospho-JNK (Cell Signaling, 4668), and ASC (Adipogen, AL177; Santa Cruz, sc-22514). After washing membranes with TBS-T for 3 × 5 min, membranes were incubated in relevant horseradish peroxidase (HRP)–conjugated secondary antibodies (Southern Biotech, 4010-05) diluted 1:5000 to 10,000 in 5% skim milk TBS-T for 1 hour at room temperature. Membranes were then washed with TBS-T for 3 × 5 min and visualized using enhanced chemiluminesence (Millipore, Bio-Rad, WBLUF0500) on the ChemiDoc Touch Imaging System (Bio-Rad).

### Tissue lysis

Mouse spleen tissues were snap frozen on dry ice and kept at −80°C. The tissue was weighed, and DISC buffer containing 20 mM tris-HCl, pH 7.5, 150 mM NaCl, 2 mM EDTA, 1% Triton X-100, 10% glycerol, H_2_O, protease inhibitor cocktail tablet (Roche, 4693132001), and Phospho-STOP phosphatase inhibitor tablet (Roche, 4906837001) was added at 10 μl/mg tissue in a 2-ml Eppendorf tube. Using metal beads, samples were homogenized with a TissueLyser 85300. Homogenized samples were incubated on ice for 20 min and then centrifuged at 20,375*g* for 15 min at 4°C. Supernatants were collected, and a bicinchoninic acid assay (Thermo Fisher Scientific, 23225) was performed to quantify protein concentrations.

### Quantitative polymerase chain reaction (qPCR)

BMDMs stimulated as indicated in the relevant figure legends were lysed in RNA lysis buffer (Meridian Bioscience, BIO-5207) and kept at −80°C. Total RNA was extracted using the ISOLATE II RNA Mini Kit (Meridian Bioscience, BIO-52073), and cDNA was generated using SuperScript III Reverse Transcriptase (Invitrogen, 18080093). Seventy nanograms of cDNA was loaded in duplicate onto plates containing 2× Maxima SYBR Green qPCR Master Mix (Thermo Fisher Scientific, K0251) with a separate ROX vial (Thermo Fisher Scientific, K0251). qPCR was performed using the ViiA 7 Real-Time PCR System (Applied Biosystems) following a protocol of 42 cycles: stage 1, 95°C for 15 min; stage 2, 94°C for 15 s, 55°C for 30 s, and 72°C for 30 s; stage 3, 95°C for 15 s, 60°C for 1 min, and 95°C for 15 s. Target gene expression was normalized to the housekeeping gene *Hprt* and plotted as the fold change (FC) relative to Hprt (Δ CT). Primer sequences used were as follows: *mm Il1b* (for.: 5′ GCTACCTGTGTCTTTCCCGT; rev.: 5′ ATCTCGGAGCCTGTAGTGC), *mm Tnf* (for.: 5′ ACTGAACTTCGGGGTGATCG; rev.: 5′ TGATCTGAGTGTGAGGGTCTGG), and *mm Nlrp3* (for.: 5′ ATTACCCGCCCGAGAAAGG; rev.: 5′ TCGCAGCAAAGATCCACACAG).

### Cytokine analysis

IL-1β (R&D, DY401), IL-6 (R&D, DY406), and TNF (R&D, DY410) enzyme-linked immunosorbent assays were performed according to the manufacturer’s protocols on serum or plasma, as indicated in the relevant figure legends.

### Blood cell analysis

Blood cell analysis was performed using the automated ADVIA 2120i hematological analyzer on blood collected via cardiac puncture (*89*).

### Histology and immunohistochemistry

Spleens were fixed in 10% neutral buffered formalin for 24 to 72 hours, then embedded in paraffin, and sectioned for histology. Immunohistochemistry sections were stained with cleaved caspase-3 antibody (CST, clone: Asp^175^, cat no. 9661, 1:300), F4/80 antibody (CST, clone: D2S9R, cat. no. 70076, 1:500), GSDMD antibody (CST, clone: E9S1X, cat. no. 39754, 1:100), caspase-1 antibody (Abcam, clone: EPR16883, ab179515, 1:100), caspase-3 antibody (CST, cat. no. 9662, 1:100), and an HRP-conjugated rabbit secondary antibody (Dako Agilent, cat. no. K400311-2, RTU) or a MACH4 universal HRP polymer (Biocare, cat. no. BRR4012L, RTU). Slides were scanned using an Olympus VS200 Slide Scanner. Hematoxylin and eosin (H&E)–stained slides were blindly evaluated by a certified tissue pathologist.

### Splenocyte isolation and preparation

Mouse spleens were collected and transferred into cold RPMI 1640 medium containing 10% FBS. Tissues were gently dissociated by passing them through a 70-μm cell strainer using a syringe plunger. The resulting suspension was centrifuged at 400*g* for 5 min at 4°C. To remove red blood cells, the pellet was treated with RBC lysis buffer [ammonium chloride, sodium bicarbonate, and EDTA (tetrasodium salt)] for 2 min at room temperature, followed by neutralization with RPMI 1640 supplemented with 10% FBS. After washing with PBS, cells were resuspended in complete RPMI 1640 medium, counted using a hemocytometer, and cultured for 24 hours in a 10% CO_2_ incubator for further experiments, as detailed in the relevant figure legends.

### RNA sequencing analysis

Bulk RNA sequencing data were obtained from the Gene Expression Omnibus database (accession number GSE183564) for subsequent analysis. Raw read counts were organized into four experimental conditions: unstimulated untreated, unstimulated JQ1-treated, LPS untreated, and LPS JQ1-treated, each with three biological replicates. Quality control assessments were performed to ensure data quality and consistent sequencing depth across samples. Lowly expressed genes were filtered out on the basis of minimum count thresholds, and the remaining counts were normalized to adjust for library size differences and improve differential expression analysis. Differential expression analysis was conducted by fitting a negative binomial model and defining specific contrasts between conditions to identify significantly differentially expressed genes (DEGs) using a false discovery rate cutoff of <0.05. The resulting DEGs were visualized with heatmaps, showing expression patterns of the top genes, and volcano plots highlighting the relationship between log_2_FCs (logFC ≥ 1 or logFC ≤ −1) and statistical significance (adjusted *P* value <0.05). In addition, a curated gene list was analyzed to highlight key gene expression changes and related biological pathways. All analyses were performed using RStudio (R version 4.2.2), with edgeR (version 3.40.2) (*90*) for differential expression analysis, ggplot2 (version 3.5.1) (*91*) and pheatmap (version 1.0.12) (*92*) for data visualization, and EnhancedVolcano (version 1.16.0) (*93*) for creating volcano plots.

### Quantification and statistical analysis

Data from all in vivo experiments were pooled from two to four independent experiments (other than *Mlkl^−/−^* mice, which were only conducted once), as indicated in the relevant figures legends, and are shown as the means ± SEM. All in vitro experiments were pooled from two to five independent biological replicates and are shown as the means ± SEM, unless stated otherwise. All immunoblots were conducted on two to four independent occasions, as indicated in the figure legends. Statistical analysis of mouse temperatures between genotypes was performed using a two-way analysis of variance (ANOVA), with a Bonferroni post hoc correction for multiple comparisons. In pooled graphs where experiments concluded at different time points, the final time point includes data only from relevant experiments. For cytokine analysis, complete blood count, and spleen weight–to–body weight analysis between two genotypes and cell death between two treatments on the same genotype, an unpaired nonparametric Mann-Whitney test was used. Survival graphs were analyzed using the log-rank (Mantel-Cox) test. All data were prepared and analyzed using GraphPad Prism (version 10.2.3). Statistical significance was set at 0.05, with *P* > 0.05 (ns), *P = 0.05, **P = 0.01, ***P = 0.001, and ****P = 0.0001.
