# Supplementary material for: NLRP3 inflammasome–driven hemophagocytic lymphohistiocytosis occurs independent of IL-1β and IL-18 and is targetable by BET inhibitors
Source: Sci Adv. 2025 Jul 9;11(28):eadv0079. doi: 10.1126/sciadv.adv0079 (PMC12239941; doi:10.1126/sciadv.adv0079)
Supplement: Supplementary file 1 — Figs. S1 to S9 Tables S1 to S3 [file sciadv.adv0079_sm.pdf]

Science Advances
AAAS

Supplementary Materials for

NLRP3 inflammasome–driven hemophagocytic lymphohistiocytosis occurs independent of IL-1β and IL-18 and is targetable by BET inhibitors

Farzaneh Shojaee *et al.*

Corresponding author: Maryam Rashidi, rashidi@wehi.edu.au; James E. Vince, vince@wehi.edu.au

Sci. Adv. **11**, eadv0079 (2025)
DOI: 10.1126/sciadv.adv0079

This PDF file includes:

Figs. S1 to S9
Tables S1 to S3

Supplementary Materials

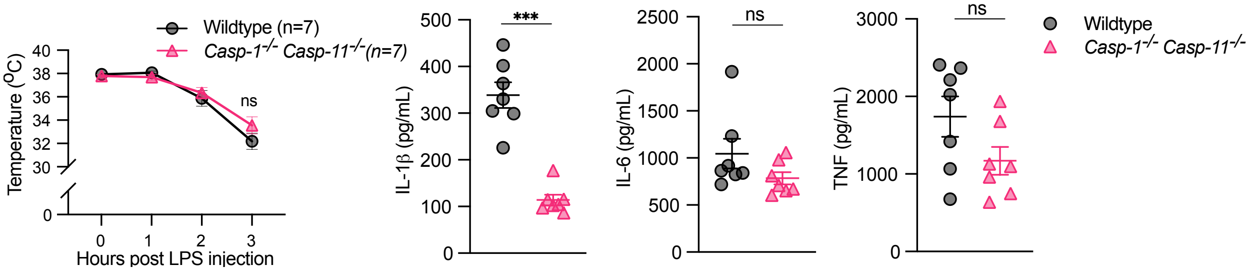

Figure S1. Caspase-1 and caspase-11 loss does not protect from sHLH-driven temperature reduction.

Rectal temperatures and serum cytokine concentrations (at 3 hrs) of wildtype (n=7) and *Casp1$^{-/-}$ Casp11$^{-/-}$* (n=7) mice following sHLH induction (data pooled from two independent experiments). Data represent the mean ± SEM. $p \leq 0.05$ (*), $p \leq 0.01$ (**), $p \leq 0.001$ (***), $p \leq 0.0001$ (****). Mouse temperatures were analyzed using a two-way ANOVA, with a Bonferroni post hoc correction for multiple comparisons, while an unpaired non-parametric Mann-Whitney test was used for analyzing cytokine levels.

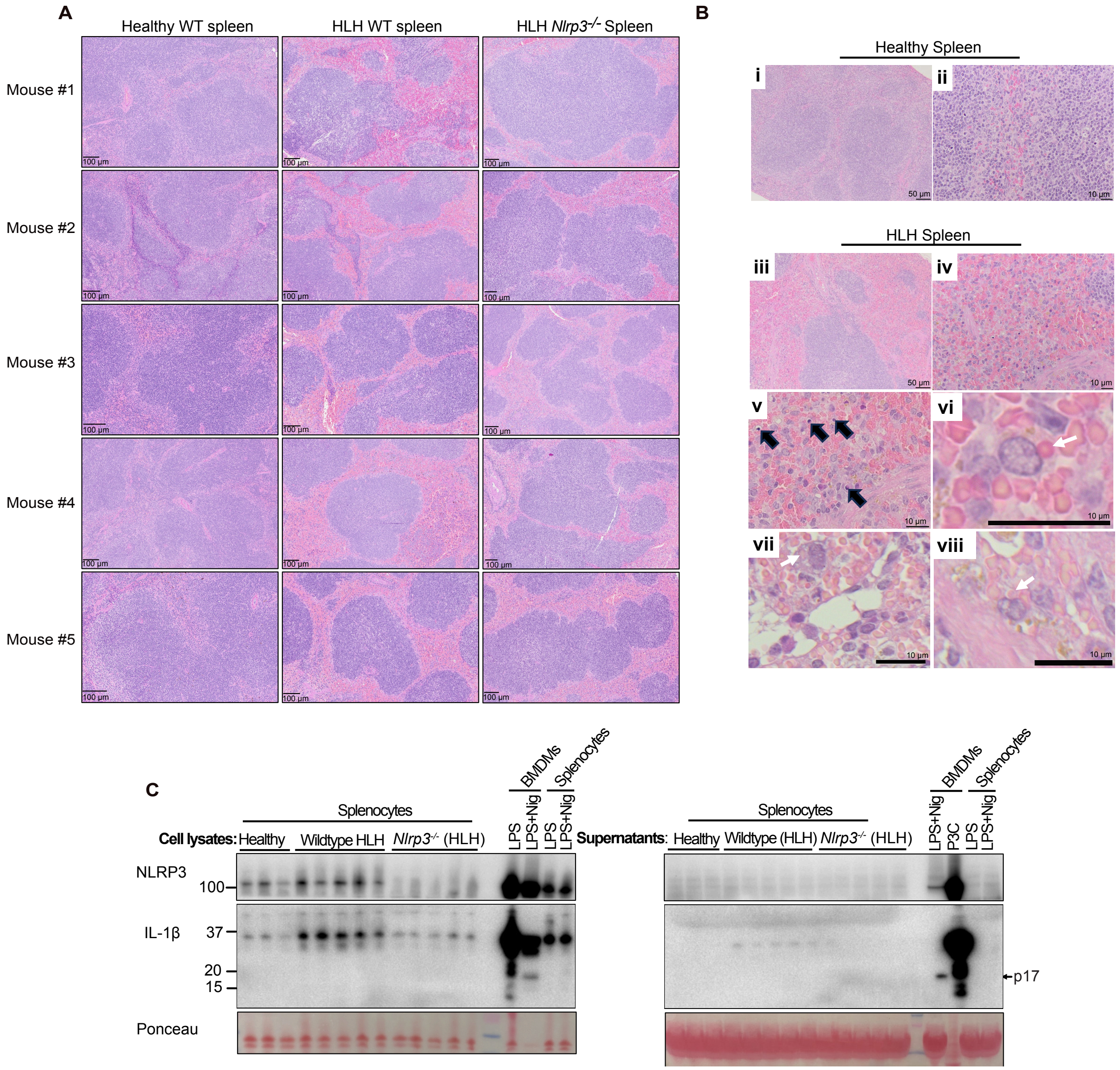

Figure S2. Deletion of NLRP3 does not alleviate sHLH-induced pathological damage in spleen tissue.

(A) H&E staining of spleen tissue harvested from WT healthy, WT HLH and *Nlrp3*$^{-/-}$ HLH 3-4 hrs after sHLH induction. Figure 1F shows mouse #5 data as a representative image.

(B) Representative (i) H&E staining of healthy spleen tissue. (ii) Higher magnification of (i), showing normal splenic architecture. (iii) Representative H&E staining of sHLH spleen tissue. (iv-vi) Higher magnifications of (iii), highlighting pathological features. Apoptotic bodies in the red pulp are indicated by black arrows, while erythrophagocytosis is marked by white arrows. (vii-viii) Erythrophagocytosis in (vii) WT sHLH spleen and (viii) *Nlrp3*$^{-/-}$ sHLH spleen.

(C) Immunoblot analysis of lysates and supernatants from *ex vivo* splenocytes isolated from WT sHLH and *Nlrp3*$^{-/-}$ sHLH spleens 3–4 hours after sHLH induction, followed by 24 hours of *in vitro* culture. BMDMs treated with LPS (100 ng/ml) or Pam3CSK4 (P3C: 500 ng/ml) and LPS and nigericin (10 μM), along with healthy splenocytes treated with LPS with or without nigericin, were used as positive controls.

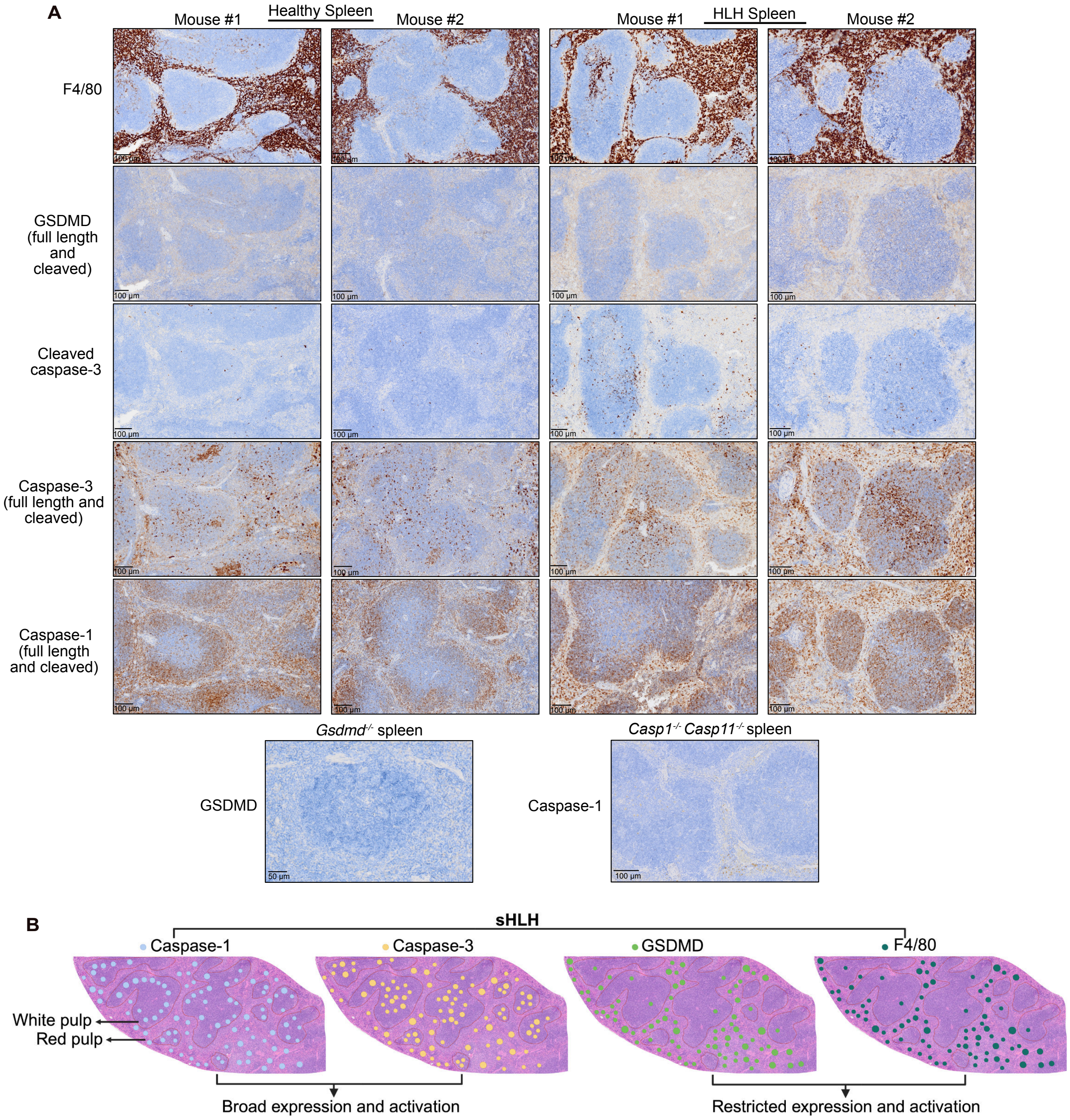

Figure S3. **GSDMD expression in the spleen is mainly restricted to F4/80⁺ macrophages, while caspase-1 and -3 are widely distributed.**

(A) Immunohistochemistry staining of healthy and sHLH spleen tissue using F4/80, GSDMD, cleaved caspase-3, caspase-3 and caspase-1 antibodies and relevant controls (spleen from Caspase-1 and -11 deficient mice and GSDMD deficient mice).

(B) Schematic depicting the expression patterns of caspase-3, caspase-1, GSDMD and macrophages in sHLH spleen tissue.

(B) created in BioRender. F. Shojaee (2025) https://BioRender.com/ljrbkb8.

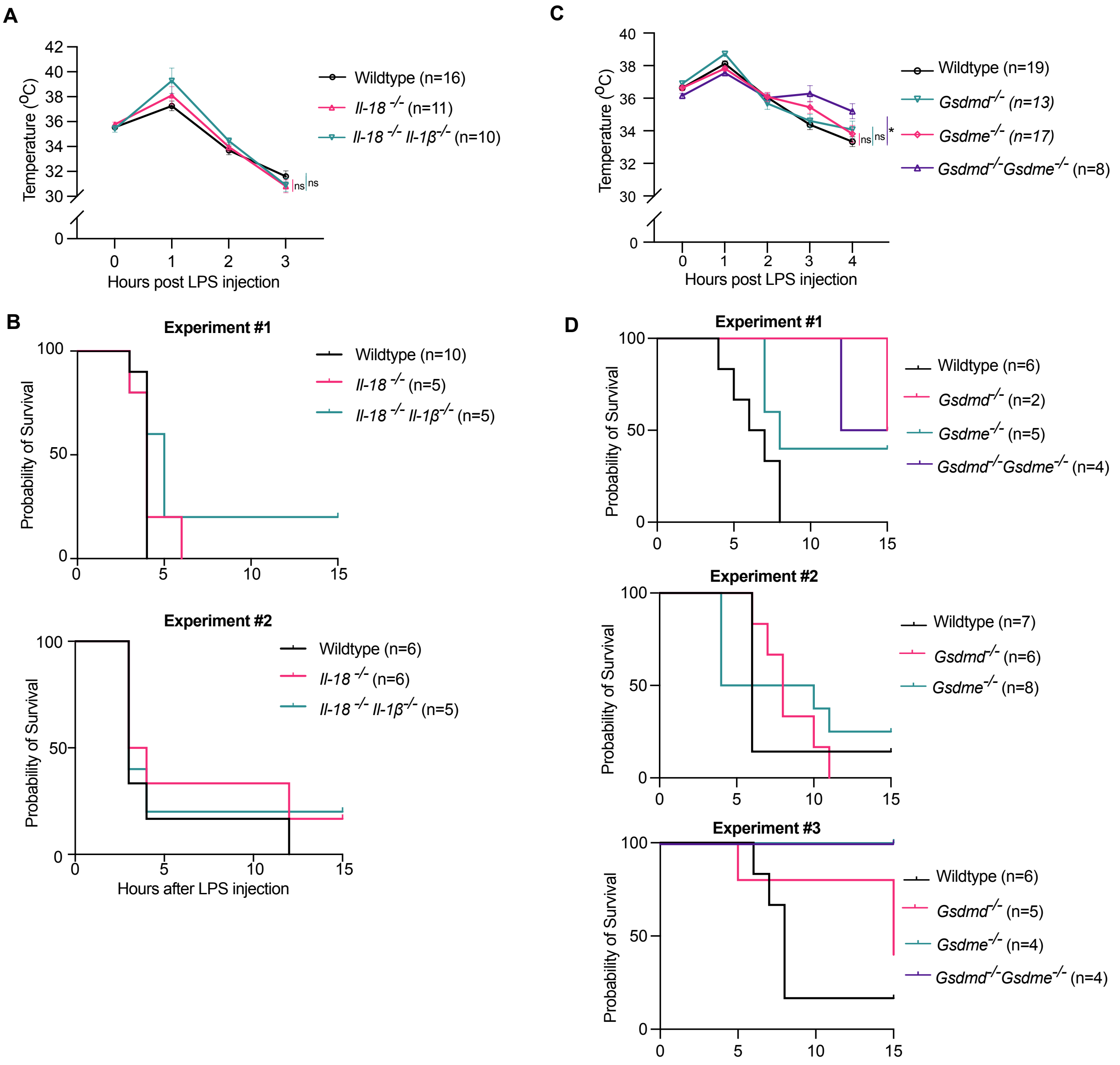

Figure S4. sHLH-induced temperature changes and survival in *Il-18$^{-/-}$Il-1β$^{-/-}$* and *Gsdmd$^{-/-}$Gsdme$^{-/-}$* mice.

(A) Rectal temperatures of wildtype (n=16), *Il-18$^{-/-}$* (n=11) and *Il-18$^{-/-}$Il-1β$^{-/-}$* (n=10) mice post sHLH induction. Data pooled from two independent experiments.

(B) Independent experiment survival curve data for the pooled analysis depicted in Fig. 2G.

(C) Rectal temperatures of wildtype (n=19), *Gsdmd$^{-/-}$* (n=13), *Gsdme$^{-/-}$* (n=17), and *Gsdmd$^{-/-}$Gsdme$^{-/-}$* (n=8) mice, post sHLH induction. Data pooled from three independent experiments.

(D) Independent experiment survival curve data for the pooled analysis depicted in Fig. 2H.

Data represent the mean value ± SEM. $p \leq 0.05$ (*), $p \leq 0.01$ (**), $p \leq 0.001$ (***), $p \leq 0.0001$ (****). Mouse temperature analysis used a two-way ANOVA, with a Bonferroni post hoc correction for multiple comparisons.

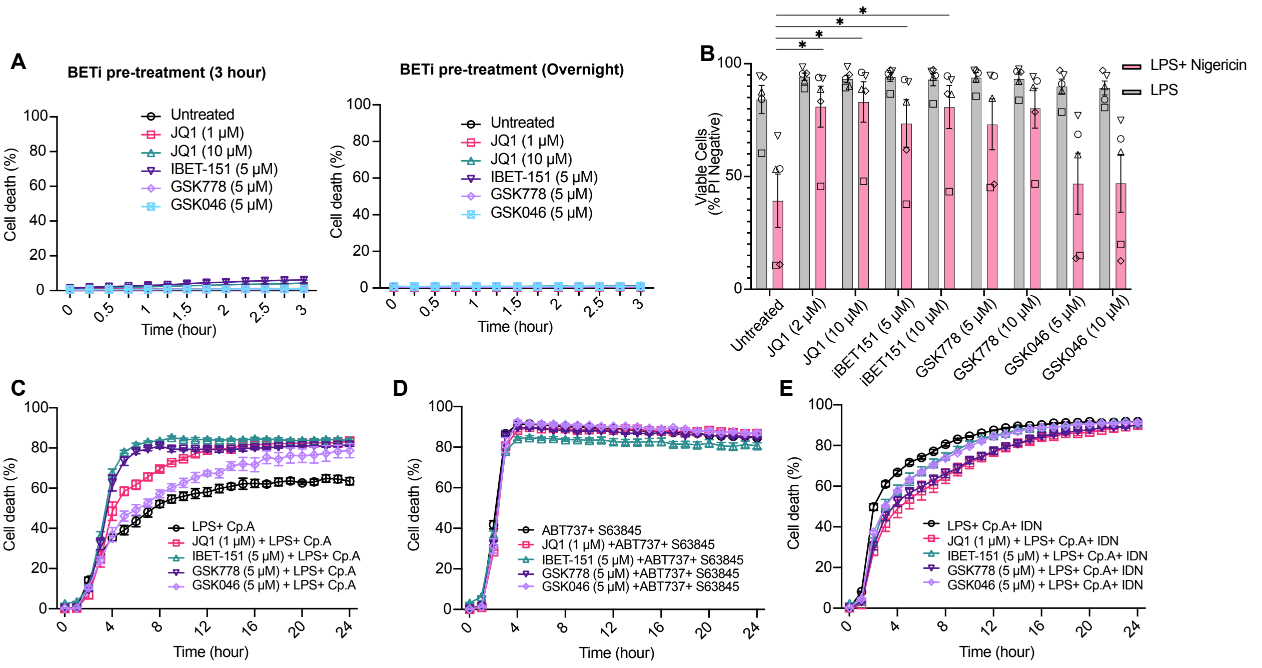

Figure S5. BET inhibitors limit NLRP3-mediated pyroptosis but do not protect macrophages from apoptosis and necroptosis.

(A) BMDMs were treated with JQ1 (1, 10 µM), iBET-151 (5 µM), GSK778 (5 µM), and GSK046 (5 µM) for 3 hrs or overnight, and cell death assessed via propidium iodide (PI) staining and live Incucyte imaging. Data is pooled from 2 independent experiments for 3-hour BETi pre-treatments, and pooled from 4 independent experiments using a total of 3 biological replicates, with one biological replicate used in two independent experiments, for overnight BETi pre-treatments.

(B) BMDMs were treated with JQ1 (2, 10 µM), iBET-151 (5, 10 µM), GSK778 (5, 10 µM), and GSK046 (5, 10 µM) overnight, then stimulated with LPS (100 ng/ml) for 3 hrs ± nigericin (10 µM) for 40 minutes. Cell death was assessed via PI uptake and flow cytometry. Data pooled from 5 independent experiments, represented by symbols.

(C, D, E) BMDMs were treated with JQ1 (1 µM), iBET-151 (5 µM), GSK778 (5 µM), and GSK046 (5 µM) overnight, and then stimulated with (**C**) LPS (100 ng/ml) and Compound A (Cp.A, 1 µM), (**D**) ABT737 (1 µM) and S63845 (10 µM), and (**E**) LPS (100 ng/ml), Cp. A (1 µM) and IDN-6556 (IDN, 10 µM). Cell death was assessed via PI uptake and live IncuCyte imaging. Data show one experiment using 3 technical replicates. Data represent the mean value ± SEM. $p \leq 0.05$ (*), $p \leq 0.01$ (**), $p \leq 0.001$ (***), $p \leq 0.0001$ (****), with statistical cell death analysis conducted using an unpaired non-parametric Mann-Whitney test.

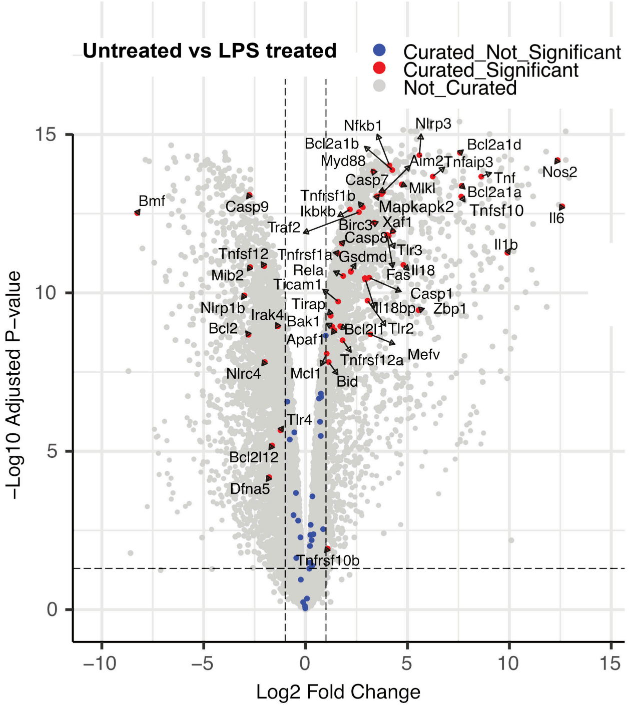

Figure S6. LPS regulation of cell death gene expression in macrophages.
A volcano plot displaying the differentially expressed genes (DEGs) associated with distinct cell death pathways in BMDMs treated with ± LPS (50 ng/ml) for 4 hrs. Adjusted p-value <0.05 and cut-off values logFC ≥1 or logFC ≤ -1 (n = 3 biological replicates). Data re-analyzed from M Hoffner O'Connor *et al.* (*54*).

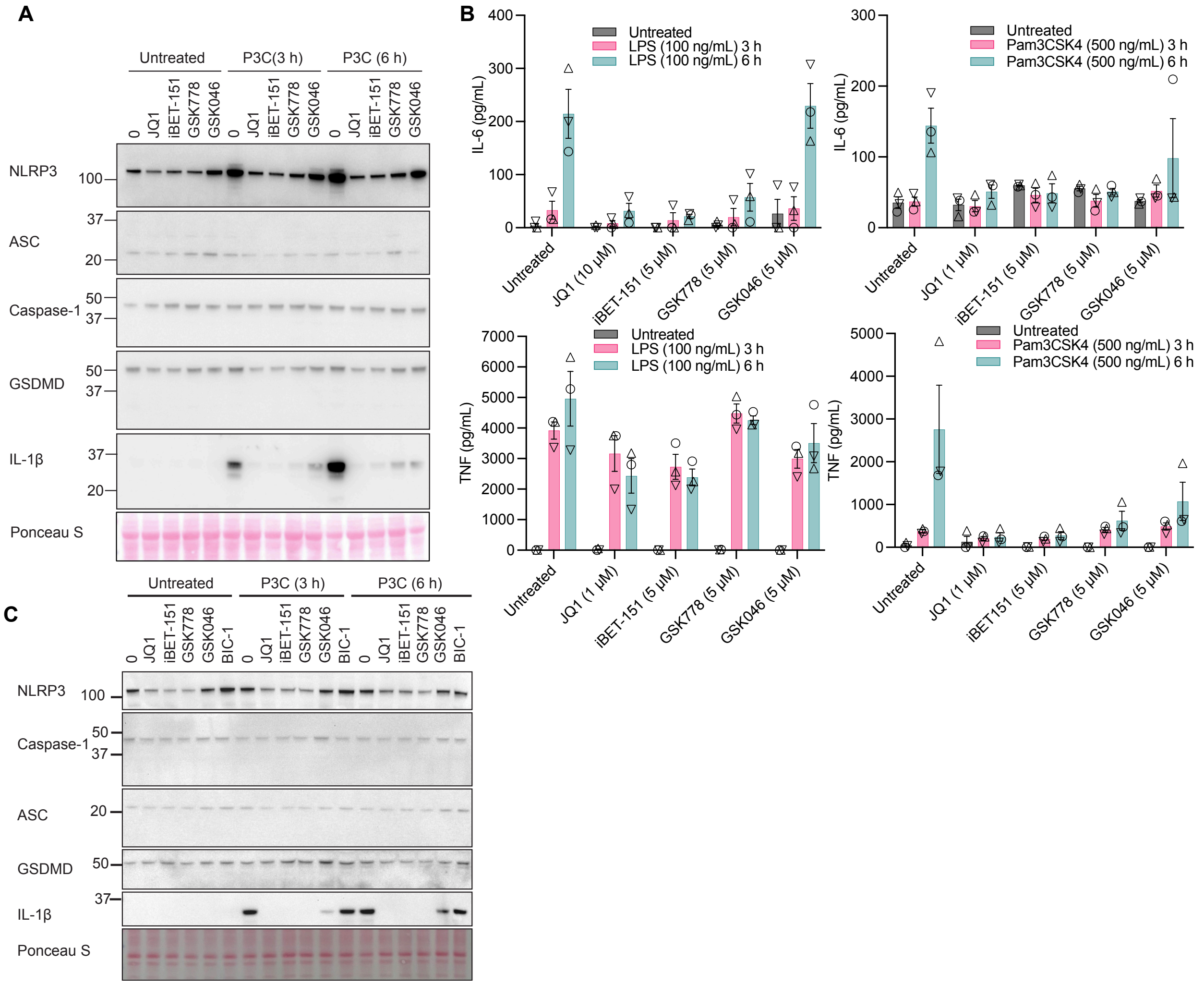

Figure S7. BET inhibitors reduce expression of NLRP3 and IL-1β in mouse and human cells.
(A) Immunoblot analysis of BMDMS treated with JQ1 (10 μM), iBET-151 (5 μM), GSK778 (5 μM), and GSK046 (5 μM) overnight then stimulated with Pam3CSK (P3C, 500 ng/ml) for 3 and 6 hrs (representative of 3 independent experiments).
(B) BMDMS were treated with JQ1 (10 μM), iBET-151 (5 μM), GSK778 (5 μM), and GSK046 (5 μM) overnight then stimulated with LPS (100 ng/ml) or Pam3CSK (P3C, 500 ng/ml) for 3 and 6 hrs. The concentration of secreted IL-6 and TNF were measured by ELISA on cell supernatants (n = 3 independent experiments, indicated by symbols).
(C) Immunoblot analysis of THP-1 cells treated with JQ1 (10 μM), iBET-151 (5 μM), GSK778 (5 μM), GSK046 (5 μM), and BIC-1 (5 μM) overnight then stimulated with Pam3CSK (P3C, 500 ng/ml) for 3 and 6 hrs (representative of 3 independent experiments).

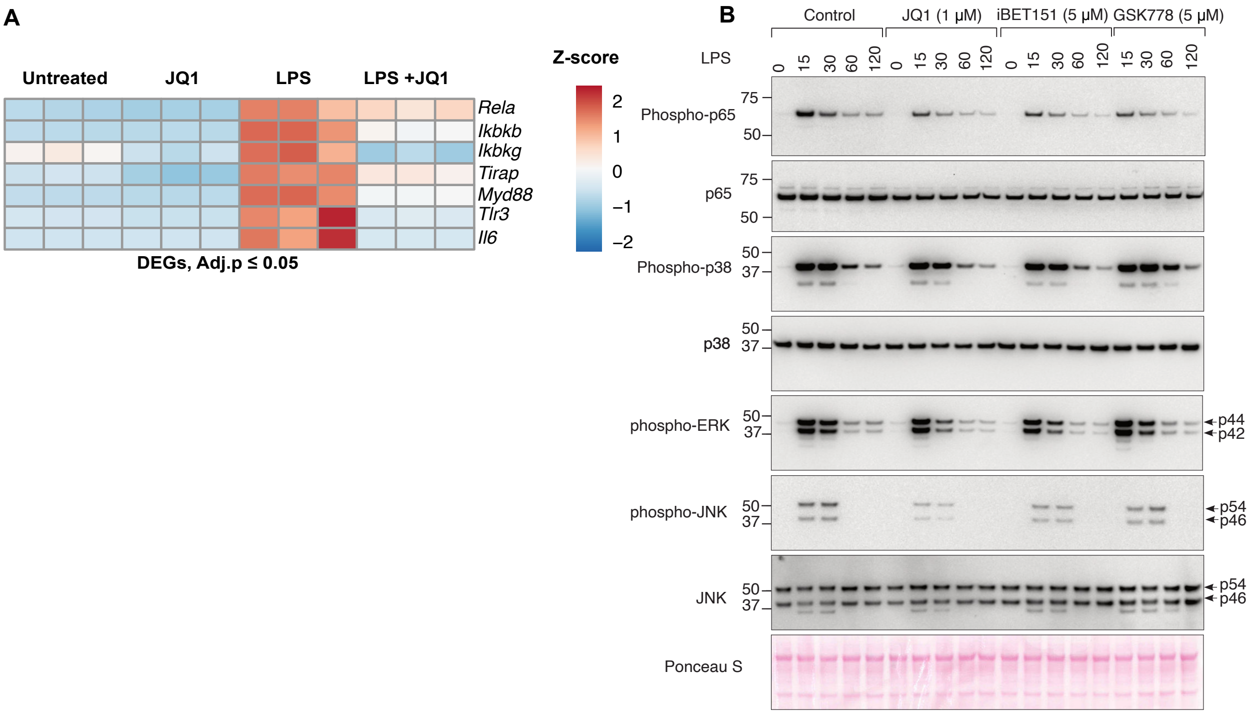

Figure S8. BET inhibitors mildly reduce NF-kB pathway activation in response to LPS.
(A) A heatmap plot of DEGs in BMDMs treated with ± JQ1 (0.5 µM) for 12 hrs followed by ± LPS (50 ng/ml) for 4 hrs. Adjusted p-value <0.05 and cut-off values logFC ≥1 or logFC ≤ -1 (n = 3 biological replicates). Data re-analyzed from M Hoffner O'Connor *et al.* (*54*).
(B) Immunoblot analysis of BMDMS treated with JQ1 (10 µM), iBET-151 (5 µM), and GSK778 (5 µM) overnight then stimulated with LPS (100 ng/ml) for 15, 30, 60, and 120 minutes (representative of 3 independent experiments).

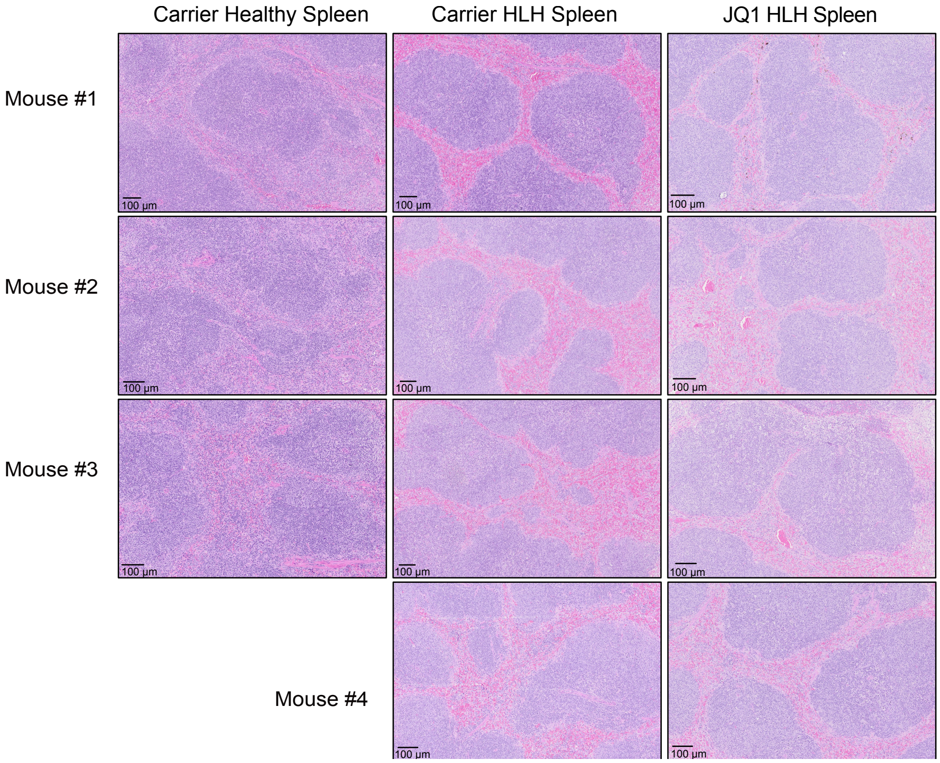

Figure S9. **JQ1 does not alter splenic pathology in sHLH.**
H&E staining of spleen tissue harvested from WT healthy, Carrier HLH and JQ1 HLH 3-4 hrs after sHLH induction. Figure 6E shows mouse #1 data as a representative image.

Supplemental Table 1. Spleen histopathology in wildtype and *Nlrp3*$^{-/-}$ mice.

	Red pulp congestion/white pulp depletion (Y/N)	Apoptotic debris (Y/N)	Overall impression abnormal (Y/N)
Healthy WT	N	N	N
Healthy WT	N	N	N
Healthy WT	N	N	N
Healthy WT	N	N	N
Healthy WT	N	N	N
HLH WT	Y	Y	Y
HLH WT	Y	Y	Y
HLH WT	Y	Y	Y
HLH WT	Y	Y	Y
HLH WT	Y	Y	Y
HLH *Nlrp3*$^{-/-}$	Y	Y	Y
HLH *Nlrp3*$^{-/-}$	Y	Y	Y
HLH *Nlrp3*$^{-/-}$	Y	Y	Y
HLH *Nlrp3*$^{-/-}$	Y	Y	Y
HLH *Nlrp3*$^{-/-}$	Y	Y	Y

Supplemental Table 2. Cell death gene categorization.

Extrinsic Apoptosis		Intrinsic Apoptosis		Pyroptosis	Necroptosis	NLRs	Others
Tnf	*Tnfaip3*	*Casp9*	*Pmaip1*	*Casp11*	*Ripk3*	*ciita*	*Tlr2*
Tnfrsf1a	*Mib1*	*Apaf1*	*Bik*	*Dfna5*	*Mlkl*	*Naip*	*Tlr3*
Tnfrsf1b	*Mib2*	*Cycs*	*Bmf*	*Gsdmd*	*Zbp1*	*Nod1*	*Tlr4*
Tnfrsf10a	*Birc4*	*Bax*	*Hrk*	*Casp1*	*Ripk1*	*Nod2*	*Rela*
Tnfrsf10b	*Cyld*	*Bak1*	*Bnip1*	*Nlrp3*		*Nlrp3*	*Nfkb1*
Tnfsf10	*Spata2*	*Bok*	*Bnip3*	*Aim2*		*Nlrc4*	*Myd88*
Fasl	*Map3k7*	*Bcl2*	*Tnfaip1*	*Mefv*			*Il1rap*
Fas	*Mapk14*	*Bcl2l1*	*Tnfaip2*	*Pycard*			*Tirap*
Tnfsf12	*Mapkapk2*	*Mcl1*	*Bnip2*	*Nlrp1b*			*Irak1*
Tnfrsf12a	*Cflar*	*Bcl2a1a*	*Casp2*	*Nek7*			*Traf6*
Ripk1	*Fadd*	*Bcl2a1b*	*Rtl10*	*Birc1*			*Ticam1*
Tradd	*Casp8*	*Bcl2a1c*	*Bak1*	*Nlrc4*			*Irf3*
Traf2	*Casp3*	*Bcl2a1d*	*Bcl2l10*	*Il1b*			*Irf7*
Birc2	*Casp6*	*Bcl2l2*	*Bcl2l12*	*Il18*			*Tbk1*
Birc3	*Casp7*	*Bcl2l11*	*Bcl2l13*	*Il18bp*			*Il10*
Rnf31	*Xaf1*	*Bid*	*Bcl2l14*	*Il18r1*			*Ikbkg*
Rbck1	*Casp10*	*Bad*	*Bcl2l15*	*Gsdma*			*Ikbkb*
Sharpin		*Bbc3*		*Gsdmb*			*Il6*
				Gsdmc			*Tram*
				Pklr			*Irak4*
							Nos2
							Hif1a

Supplemental Table 3. Spleen histopathology in wildtype and JQ1 treated mice.

	Red pulp congestion/white pulp depletion (Y/N/Equivical)	Apoptotic debris (Y/N/Equivical)	Overall impression abnormal (Y/N)
Carrier Healthy	N	N	N
Carrier Healthy	N	N	N
Carrier Healthy	N	N	N
Carrier HLH	Y	Y	Y
Carrier HLH	Y	Y	Y
Carrier HLH	Y	Y	Y
Carrier HLH	N	N	Y
JQ1 HLH	E	E	Y
JQ1 HLH	Y	Y	Y
JQ1 HLH	E	E	Y
JQ1 HLH	Y	Y	Y
